# Electric-Field-Induced Connectivity Switching in Single-Molecule Junctions

**DOI:** 10.1016/j.isci.2019.100770

**Published:** 2019-12-14

**Authors:** Chun Tang, Jueting Zheng, Yiling Ye, Junyang Liu, Lijue Chen, Zhewei Yan, Zhixin Chen, Lichuan Chen, Xiaoyan Huang, Jie Bai, Zhaobin Chen, Jia Shi, Haiping Xia, Wenjing Hong

**Affiliations:** 1 State Key Laboratory of Physical Chemistry of Solid Surfaces, College of Chemistry and Chemical Engineering, Collaborative Innovation Center of Chemistry for Energy Materials, Xiamen University, 361005 Xiamen, China

**Keywords:** Molecular Electrochemistry, Quantum Electronics, Electronic Materials

## Abstract

The manipulation of molecule-electrode interaction is essential for the fabrication of molecular devices and determines the connectivity from electrodes to molecular components. Although the connectivity of molecular devices could be controlled by molecular design to place anchor groups in different positions of molecule backbones, the reversible switching of such connectivities remains challenging. Here, we develop an electric-field-induced strategy to switch the connectivity of single-molecule junctions reversibly, leading to the manipulation of different connectivities in the same molecular backbone. Our results offer a new concept of single-molecule manipulation and provide a feasible strategy to regulate molecule-electrode interaction.

## Highlights

• A strategy to *in-situ* switch the connectivity of single-molecule junctions
• A concept to manipulate the molecule-electrode interaction
• A molecular switch triggered by the varying of electric field
• Experiments were combined with calculations to probe the switching mechanism

## Graphical Abstract

**Figure:**
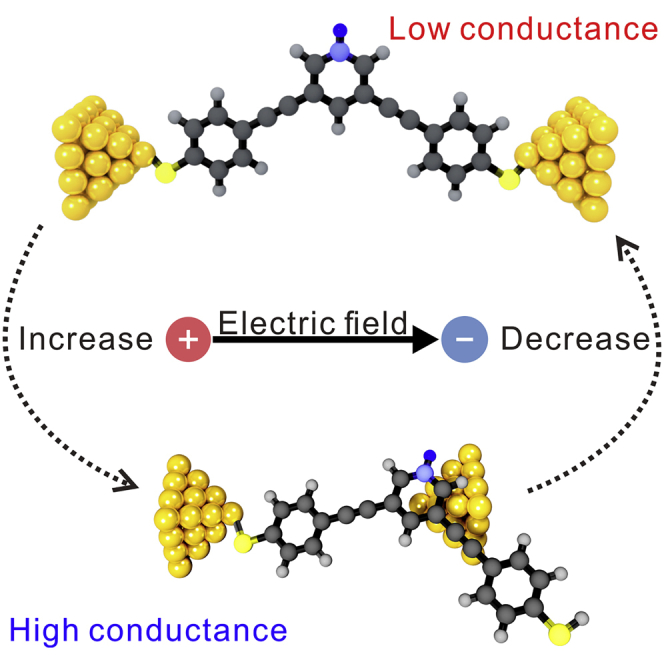

## Introduction

The interaction between molecular components and electrodes is of fundamental importance to fabricate molecular devices (Hines et al., 2013, Moth-Poulsen and Bjørnholm, 2009, Ratner, 2013, Su et al., 2016, Xiang et al., 2016a). Pre-setting anchor groups (such as pyridine and thiol) in molecular backbones is one of the most typical strategies to manipulate the molecule-electrode interaction, which links the molecules to electrodes in designed connectivity (Leary et al., 2015). The connectivity of molecular devices determines not only the pathways of charge transport through molecule backbones but also the electronic properties of the molecule devices (Lambert, 2015, Liu et al., 2019). Such as the benzene in *meta*- and *para*-connectivity shows different types of quantum interference, which leads to significantly different conductance (Agraït et al., 2003, Aradhya et al., 2012b, Arroyo et al., 2013, Bai et al., 2019, Ballmann et al., 2012, Carlotti et al., 2018, Darwish et al., 2012, Frisenda et al., 2016, Garner et al., 2018, Guedon et al., 2012, Li et al., 2017, Li et al., 2019, Liu et al., 2019, Mayor et al., 2003, Solomon et al., 2010, Su et al., 2016, Tang et al., 2019, Thompson and Nijhuis, 2016, Xiang et al., 2016a, Yoshizawa et al., 2008). The connectivity of single-molecule junctions can also determine the coupling site from the electrode to the molecule component, which has been utilized to construct a molecular switch by mechanical control (Aradhya et al., 2012a, Meisner et al., 2012, Quek et al., 2009). Moreover, such connectivity can regulate the coupling between electrodes and functional units of molecular components, which is essential for the design of molecular devices (Chen et al., 2017, Mayor et al., 2003, Xiang et al., 2016b). Because of the importance of connectivity in molecule devices, intensive efforts have been paid to construct stable and specific connectivity, whereas the manipulation of such connectivity in the same molecule backbone remained technically challenging. However, the efforts to reversibly tune the connectivity in the same molecular backbone would arouse new strategy to regulate the molecule-electrode interaction and lead to molecular devices with unique performances.

**Figure 1:**
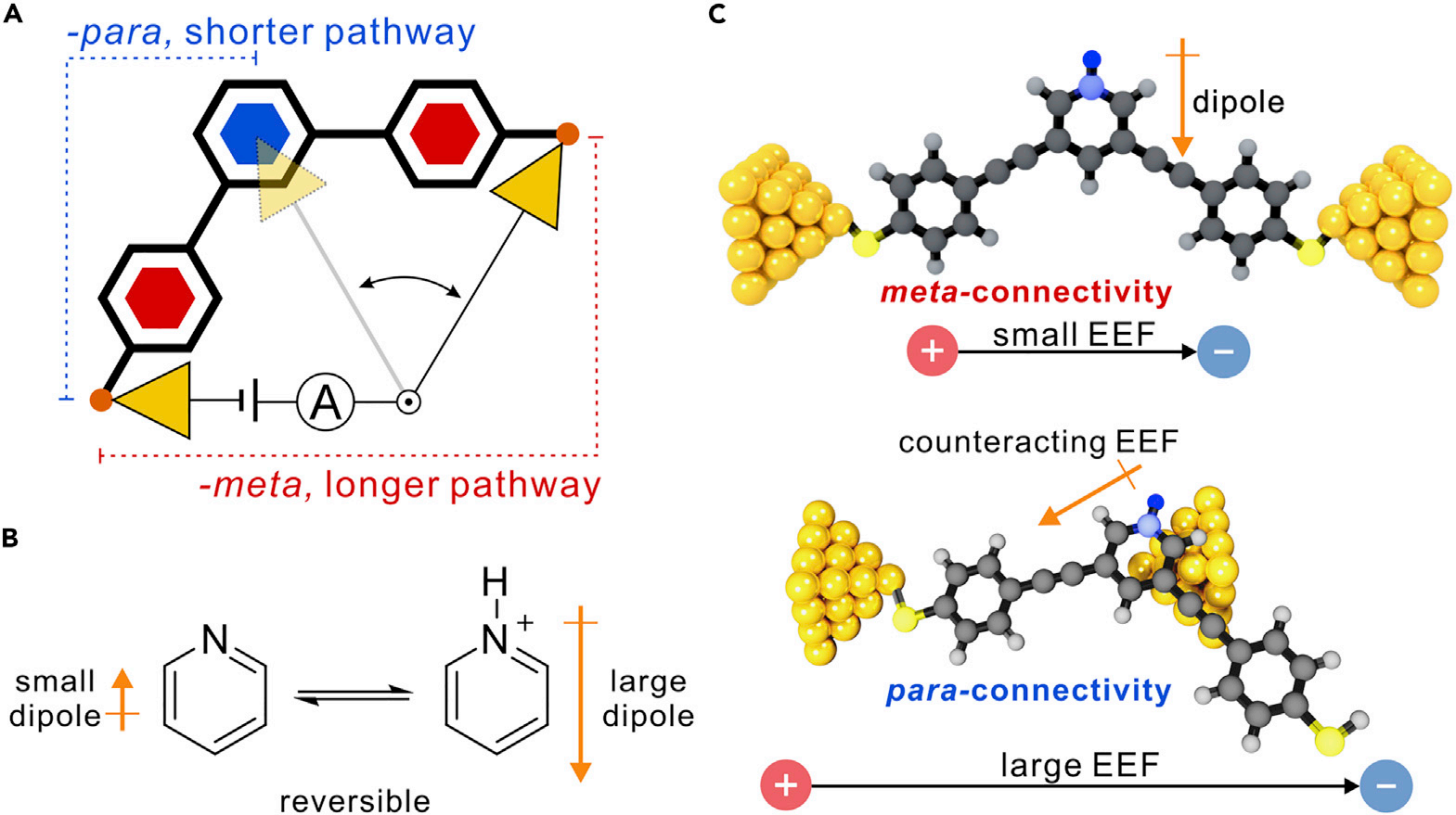
A Single-Molecule Device Based on Connectivity Switching (A) Schematics of single-molecule switch modulated by connectivity switching. The *meta*-connectivity is associated to a longer transmission pathway with low conductance, whereas the *para*-connectivity is associated to a shorter transmission pathway with high conductance. (B) The protonation of pyridine leads to a significantly enhanced dipole moment in pyridinium. (C) Schematics of electric-field-induced connectivity switching between *meta*- and *para*-connectivity. The *para*-connectivity is expected to be favorable when large EEF applied, owing to the counteracting of dipole moments with EEF. See also Figures S11, S25, and S27.

Recently, external electric field (EEF) has been proved to be a powerful tool to alter charge state (Koren et al., 2016), rupture chemical bonds (Zhang et al., 2018), vary molecule conformations (Bi et al., 2018, Gerhard et al., 2017, Lörtscher et al., 2006, Meded et al., 2009, Meng et al., 2019, Olavarria-Contreras et al., 2018), and even catalyze chemical reactions at the single-molecule scale (Aragonès et al., 2016, Ciampi et al., 2018, Huang et al., 2019, Shaik et al., 2016, Shaik et al., 2018, Wang et al., 2018). The interaction between molecular components and EEF is based on the dipole-dipole interaction. Thus the tuning of such interaction provides the opportunity to regulate the favorable connectivity of single-molecule junctions in a neat and reversible way. To achieve such a goal, we choose pyridine as the functional building block. Pyridine can be protonated with significantly enhanced dipole moments (Figure 1B), which would prefer to reorient itself to counteract EEF, with the increasing trend to form an antiparallel arrangement when the strength of EEF increased (Figure 1C) (Brooke et al., 2018, Fujii et al., 2015, Li et al., 2016, Vergeer et al., 2006). Meanwhile, pyridine also has the binary interaction with electrodes by the ring coupling or the lone pair coordination (Aradhya et al., 2012a, Quek et al., 2009), providing a potential anchor to form the in-backbone connectivity (Miguel et al., 2015). Thus, the introduction of EEF into pyridine-based molecular devices provides a promising platform toward the regulating of two possible connectivities in the same molecular skeletons.

In this work, we find that the ring of pyridinium could interact with the gold electrode, so we place pyridine in the middle of the molecular skeletons to set the two possible connectivities: the end-to-end *meta*-connectivity and the in-backbone *para*-connectivity (Figures 1A and 1C). We find that the formation of the two connectivities is controlled by protonation and the applied bias between two electrodes, suggesting that the interaction between dipole moments and the electric field is essential to tune the connectivities of single-molecule junctions. Moreover, the switching between *meta*- and *para*-connectivity is associated with the changing of transport distances from longer to shorter transmission pathways, which enlarge the conductance difference in two connectivities. Utilizing this strategy, we reversibly switch the connectivities in the same molecular skeleton and provide a new concept to efficiently manipulate single-molecule junctions.

## Results

### Single-Molecule Conductance Measurement

**Figure 2:**
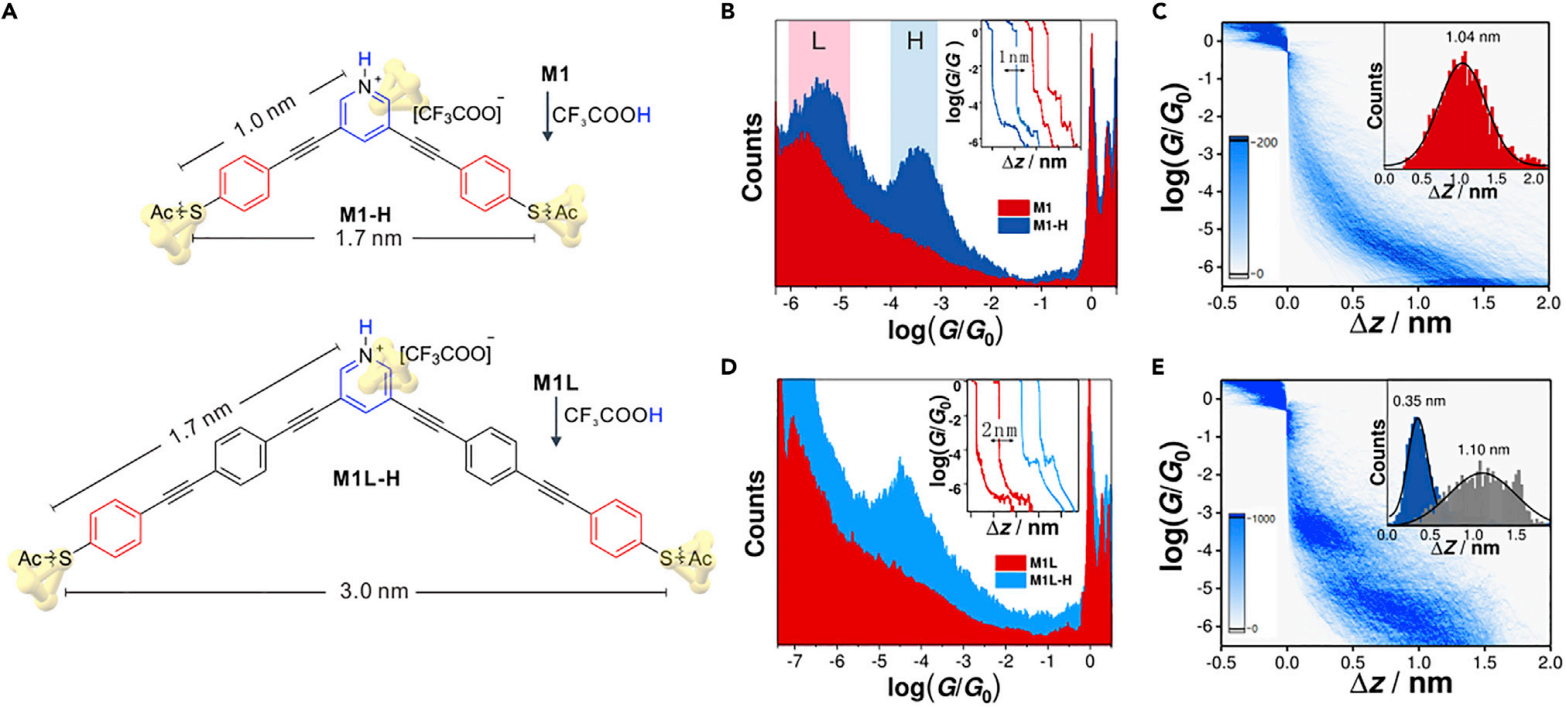
Single-Molecule Conductance Measurement (A) Molecular structures of **M1-H** and **M1L-H**, which are formed by the protonation of the neutral state **M1** and **M1L** by TFA. The calculated junction lengths for the *meta*- and *para*-connectivity are shown beside. (B–E) (B) All data-point one-dimensional conductance histograms constructed from 2,000 MCBJ traces of **M1** and **M1-H**. The typical individual traces of **M1** and **M1-H** are shown in the inset. The high- and low-conductance junctions are labeled by “H” and “L” in the blue and red region, respectively. Two-dimensional conductance histograms of **M1** (C) and **M1-H** (E) with stretching distance Δ*z* distributions shown inset. The blue and gray histograms represent the stretching distances of high- and low-conductance junctions of **M1-H**, respectively. (D) All data-point one-dimensional conductance histograms constructed from about 1,000 MCBJ traces of **M1L** and **M1L-H**, respectively. The above measurements were performed in the solvent mixture of TCB/DCM (*v*/*v*, 4/1) at room temperature with 0.10 V bias applied. See also Figures S1–17, S22, and S23.

Protonated pyridinium **M1-H** was formed *in-situ* by adding trifluoroacetic acid (TFA) to the solution of **M1** (Figure 2A), which is the neutral state of **M1-H**. The single-molecule conductances are characterized by mechanically controllable break junction (MCBJ) technique (Hong et al., 2012, Li et al., 2017) in the solvent mixture of 1,2,4-trichlorobenzene (TCB)/dichloromethane (DCM). As shown in the inset of Figure 2B, the conductances of single-molecule junctions were recorded during repeated connecting and breaking of two gold electrodes, leading to the individual traces of conductance (on the logarithmic scale) versus stretching distance (Δ*z*). The one-dimensional (1D) conductance histograms of **M1** (blue) and **M1-H** (red) are constructed from ∼2000 of such traces. As shown in Figure 2B, the sharp peaks at *G*_0_ represents the formation of gold atomic point contact (Yanson et al., 1998), and the broader peaks are associated to the conductance of corresponding single-molecule junctions, whereas the control experiments in the blank solvent did not show such signal (Supplemental Information, Figure S12). We find that **M1** shows a mono conductance peak, with the most probable conductance at 10^−5.8^ *G*_0_ (Figure 2B), which is consistent with the previous result with the presence of destructive quantum interference (Liu et al., 2017). Differently, **M1-H** shows two distinct conductance peaks (10^−3.5^ and 10^−5.4^ *G*_0_), suggesting the formation of two types of junction geometries, with about two orders of magnitude conductance difference.

The two-dimensional (2D) conductance-displacement histogram of **M1-H** (Figure 2E) demonstrates that the high-conductance junctions have about 0.35 nm stretching distance, which is significantly shorter than the low-conductance junctions of **M1** with a 1.04 nm stretching distance around 10^−6.0^ *G*_0_ (Figure 2C). The significantly shorter stretching distance for the high-conductance junction of **M1-H** is associated to the junction geometry formed between one of the –SAc groups and the middle pyridinium ring (Figure 1B), which was confirmed by a series of reference experiments (Supplemental Information, Section 3, Figures S16 and S17). Although pyridine is not a good candidate to form the in-backbone connectivity (Liu et al., 2017, Miguel et al., 2015), the *in-situ* formed pyridinium is feasible to form the in-backbone connectivity. We think such feasibility is associated with the significantly enhanced dipole moments in pyridiniums (Figure S25A), which would have a stronger interaction with the electric field applied by the two electrodes, playing an essential role in favoring the formation of the high-conductance junctions in **M1-H**. Meanwhile, the features of single-molecule conductance between **M1** and **M1-H** could be reversibly emerged when acid or base added (Figure S20).

The strategy to tune the connectivity of single-molecule junctions offers the chance to further enhance the conductance difference between the low- and high-conductance junctions, by enlarging the difference of charge transport distances in between (Cheng et al., 2011, Choi et al., 2008, Dell et al., 2015). Toward this goal, we designed molecules **M1L-H** formed by the protonation of **M1L**, leading to a 1.3 nm difference between two possible connectivities (Figure 2A), which is almost two-fold than that in **M1-H** (Figure S23). As shown in Figure 2D, **M1L** shows a mono conductance peak at 10^−7.1^ *G*_0_, attributing to the end-to-end *meta*-connectivity (Figure S22). The conductance peak for the protonated **M1L-H** locates at 10^−4.5^ *G*_0_, attributing to the high-conductance junctions, whereas the low-conductance junctions of **M1L-H** have the conductance below detecting limit, suggesting that the conductance difference between the two connectivities in **M1L-H** is increasing to ∼400 times. The results suggest that the manipulation of the difference of charge transport distances would lead to larger conductance difference in the two connectivities. More importantly, the conductance difference can be fine-tuned and further increased by this strategy, but the quantitative investigation of a molecular system with even more substantial conductance difference is restricted by the detecting limit of single-molecule conductance measurement.

### Revealing the Role of the Electric Field

**Figure 3:**
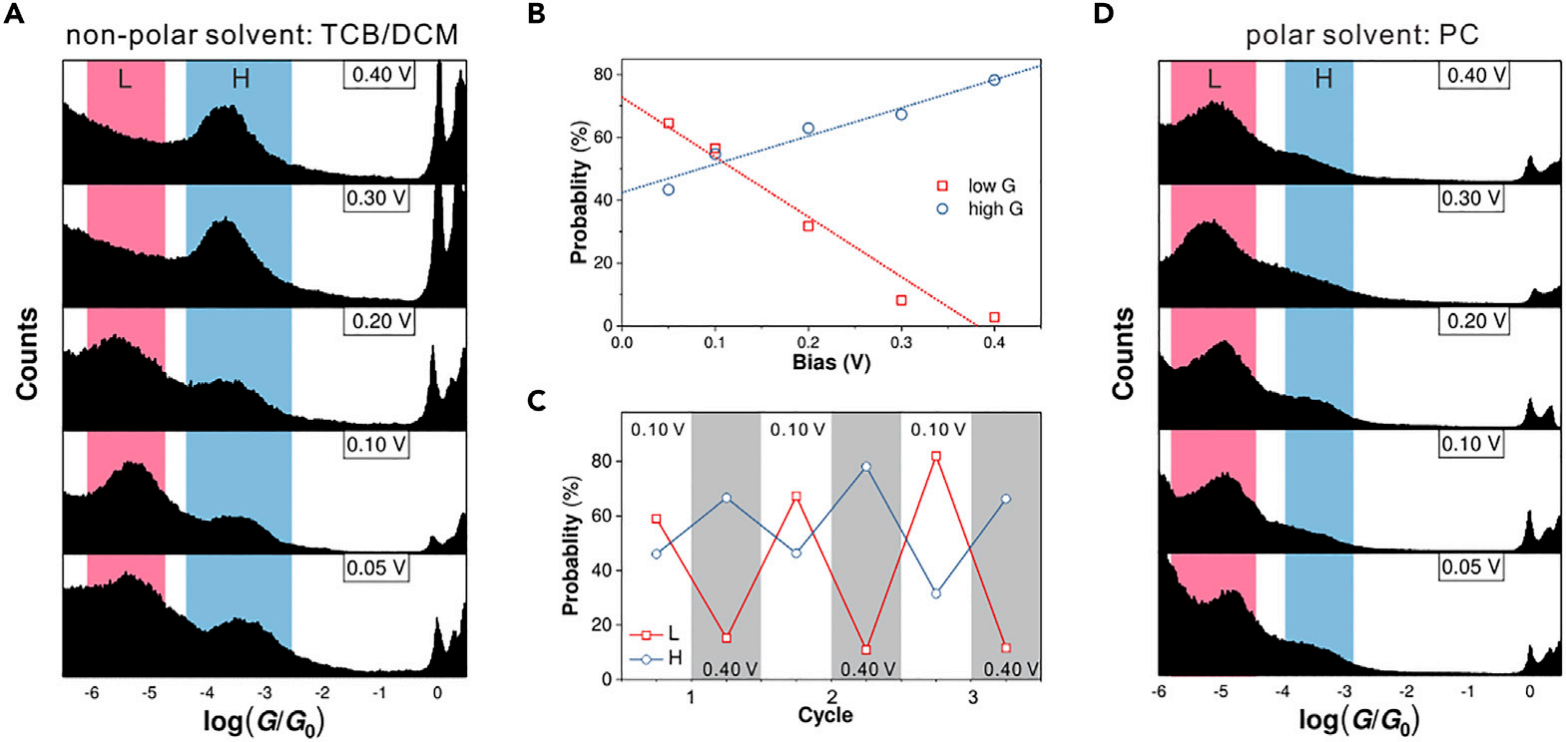
Bias-Dependent Junction Formation Probability (A) One-dimensional conductance histograms of **M1-H** with a different bias applied, in the solvent TCB/DCM mixture (*v*/*v*, 4/1). (B) The junction formation probability of **M1-H** for the corresponding low- and high-conductance junctions, respectively are labeled by the blue and red dashed lines plotted by the linear fitting. (C) The junction formation probability for the low- and high-conductance junctions of **M1-H** with 0.10 and 0.40 V bias applied alternately. (D) One-dimensional conductance histograms of **M1-H** with a different bias applied, in the solvent of propylene carbonate (PC). The above measurements were performed at room temperature. See also Figures S17–S19, S21, S24, and S26.

To understand the interaction between the molecular component and the electric field, we varied the bias voltages applied to the molecular junctions in the single-molecule conductance measurement. On account of the detecting limit, we focused the investigation on **M1-H**. Firstly, by increasing the bias from 0.05 to 0.40 V for the MCBJ measurement of **M1-H** in a nonpolar solvent (TCB/DCM), as shown in Figure 3A, we find that the formation of low-conductance junctions in **M1-H** is gradually suppressed and almost completely suppressed in the bias of 0.40 V. Meanwhile, the formation of high-conductance junctions in **M1-H** becomes more and more favorable with the increasing of bias. We quantitatively characterize the junction formation probability for both the low- and high-conductance junctions of **M1-H** in different bias (Figure S18); as shown in Figure 3B, we find that the low-conductance junctions are dominant in 0.05 V bias, whereas the high-conductance junctions become dominant when the bias is higher than 0.20 V. From the overall trend, the junction formation probability for the high-conductance junctions of **M1-H** has a positive correlation to the bias, which has a negative correlation to the low-conductance junctions of **M1-H**. Moreover, when the bias is switched between 0.10 and 0.40 V, as shown in Figure 3C, we find the high- and low-conductance junctions of **M1-H** become dominated alternately in a reversible way (Figure S19).

To further reveal the role of the electric field, we use a polar solvent, propylene carbonate (PC), to characterize the single-molecule conductance of **M1-H**. As shown in Figure 3D, the high-conductance junctions of **M1-H** are significantly suppressed even in higher bias. We also find that such bias-dependent junction formation probability of **M1-H** observed in nonpolar solvent also vanished in the polar solvent. In consideration of the changing of the equilibrium between **M1** and **M1-H** when we use PC, a polar solvent showing weak basicity, we also characterized the response of the methylated pyridinium of **M1** to electric field (Figure S17). We also find that the bias-dependent suppression of the low conductance junctions in non-polar solvent (Figure S17D) also vanish in polar solvent (Figure S17E), suggesting the importance of the dielectric constant in tuning such electric-field-induced connectivity switching. Because the polar solvent results in the attenuation of the electric field (Bermudez et al., 2000), the absence of high-conductance junctions in **M1-H** suggests the importance of the electric field to regulate the connectivities of single-molecule junctions.

### Theoretical Calculations

**Figure 4:**
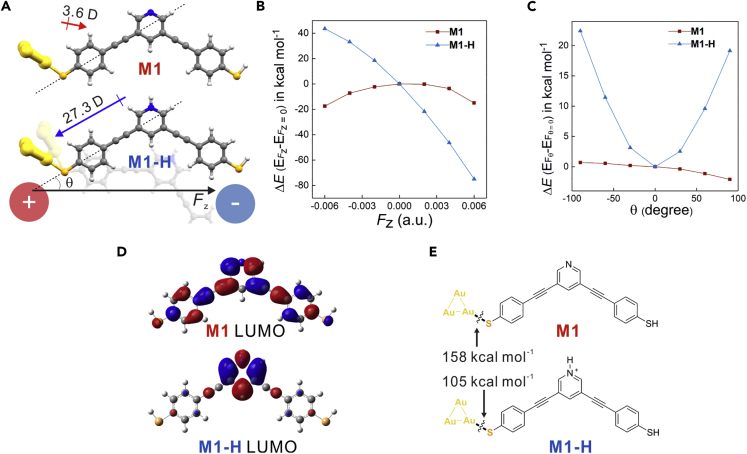
Theoretical Calculation (A) The strength and direction of dipole moments for **M1** and **M1-H** were shown by the red and blue arrows nearby; the angle between molecule orientation (dash line) and applied electric field *F*_z_ was defined as θ. Symbol D represents Debye, the unit of dipole moments. (B) Plots of total energy difference Δ*E* (E_*F*z_ - E_*F*z = 0_) versus the applied electric field when θ = 0. (C) Plots of total energy difference Δ*E* (E_θ_ - E_θ = 0_) versus θ, with electric field *F*_z_ = + 0.002 a.u. applied. (D) The orbital isosurfaces of LUMOs of **M1** and the cation of **M1-H**. (E) The Au-S covalent bonds formation energy of **M1** and **M1-H**. See also Figures S21 and S24.

To investigate the connectivity switching mechanism in **M1-H**, we carried density functional theory (DFT) calculation to study the different binding geometries between **M1** and **M1-H**. We find that the dipole moment of **M1-H** is eight times larger than **M1** (Figure 4A), attributing to the net positive charge in **M1-H** (Figure S25). The models with one of the sulfur binding to the gold electrode are used for analyzing. The effect of EEF was evaluated by the total energy changing versus the strength of EEF and the relative orientation between EEF and molecules (Figure 4A dash line). As shown in Figure 4B, fixing the EEF paralleled to the dashed line (θ = 0), with the strength of EEF changing from −0.006 to 0.006 a.u. (−3.1 to 3.1 V/nm), the total energy of **M1-H** varies about 120 kcal mol^−1^, whereas such an effect for **M1** is negligible. Upon changing θ from −90° to 90° with fixed EEF strength (+0.002 a.u.), as shown in Figure 4C, the most favorable molecular orientation for **M1-H** is the in-backbone connectivity (θ = 0) with a parallel orientation to EEF, whereas **M1** does not show explicit dependency to θ. The calculation result is consistent with the bias-dependent junction formation probability, in which the in-backbone connectivity of **M1-H** becomes more and more dominant in higher bias (Figure 3A). Besides the difference of dipole moments between **M1** and **M1-H**, the electrostatic potential distributions of **M1-H** shows significantly high positive charge distribution around the pyridinium ring (Figure S25B), so that the electrostatic attraction between the electrode and the pyridinium ring of **M1-H** would be another factor in facilitating the formation of high-conductance junctions in **M1-H**.

We also find that the formation of pyridinium has a distinct effect on their frontier orbitals. As shown in Figure 4D, the LUMO of **M1-H** is localized at the pyridinium ring, which is distinct to **M1** with its LUMO delocalized around the molecular skeleton. The localized LUMO of **M1-H** weakens the back-donating bonding from gold to sulfur, leading to weaker Au-S bond, which is confirmed by DFT calculation (Figure 4E) and surface-enhanced Raman spectra that the vibration mode of Au-S was red-shifted from 249 cm^−1^ in **M1** to 234 cm^−1^ in **M1**-**H** (Figure S21) (Kocharova et al., 2007). The weaker Au-S bond in **M1-H** reduces the competition to form the end-to-end connectivity between two sulfurs and makes the formation of the in-backbone connectivity more favorable. Thus, we think both the electric field and the weakened Au-S bonds contribute to the formation of high-conductance junctions in **M1-H**.

## Discussion

In conclusion, we have developed an electric-field-induced strategy for reversible switching the connectivities of single-molecule junctions. Through the switching from longer *meta*-connectivity to shorter *para*-connectivity, we manipulate the charge-transport distances, which significantly enhance the conductance difference between two connectivities. The mechanism of the switching is further investigated by experiments and DFT calculation, revealing that the protonation-enhanced dipole moments have significant interaction with the electric field, which favors the formation of in-backbone *para*-connectivity. Our studies suggest that the interplay between the dipole moment of molecules and EEF will lead to a reversible connectivity switching strategy, which would provide a new concept to manipulate the molecule-electrode interaction and be promising for constructing new conceptual molecular devices.

### Limitations of the Study

The switching from the end-to-end connection to the in-backbone connection of **M1-H** may also lead to the switching of quantum interference in the charge transport through the single-molecule junctions. For instance, the changes from *meta*-connection to *para*-connection may switch the patterns of quantum interference from destructive to constructive states and also offer a new opportunity for interference-based molecular devices. However, the understanding of quantum interference patterns needs further investigations, which are challenging to be accomplished at the current stage.

## Methods

All methods can be found in the accompanying Transparent Methods supplemental file.

## Supplemental Information

Document S1. Transparent Methods and Figures S1–S28

## References

[bib1] Agraït N., Yeyati A.L., van Ruitenbeek J.M. (2003). Quantum properties of atomic-sized conductors. Phys. Rep..

[bib2] Aradhya S.V., Frei M., Hybertsen M.S., Venkataraman L. (2012). Van der waals interactions at metal/organic interfaces at the single-molecule level. Nat. Mater..

[bib3] Aradhya S.V., Meisner J.S., Krikorian M., Ahn S., Parameswaran R., Steigerwald M.L., Nuckolls C., Venkataraman L. (2012). Dissecting contact mechanics from quantum interference in single-molecule junctions of stilbene derivatives. Nano Lett..

[bib4] Aragonès A.C., Haworth N.L., Darwish N., Ciampi S., Bloomfield N.J., Wallace G.G., Diez-Perez I., Coote M.L. (2016). Electrostatic catalysis of a diels–alder reaction. Nature.

[bib5] Arroyo C.R., Tarkuc S., Frisenda R., Seldenthuis J.S., Woerde C.H.M., Eelkema R., Grozema F.C., van der Zant H.S.J. (2013). Signatures of quantum interference effects on charge transport through a single benzene ring. Angew. Chem. Int. Ed..

[bib6] Bai J., Daaoub A., Sangtarash S., Li X., Tang Y., Zou Q., Sadeghi H., Liu S., Huang X., Tan Z. (2019). Anti-resonance features of destructive quantum interference in single-molecule thiophene junctions achieved by electrochemical gating. Nat. Mater..

[bib7] Ballmann S., Härtle R., Coto P.B., Elbing M., Mayor M., Bryce M.R., Thoss M., Weber H.B. (2012). Experimental evidence for quantum interference and vibrationally induced decoherence in single-molecule junctions. Phys. Rev. Lett..

[bib8] Bermudez V., Capron N., Gase T., Gatti F.G., Kajzar F., Leigh D.A., Zerbetto F., Zhang S. (2000). Influencing intramolecular motion with an alternating electric field. Nature.

[bib9] Bi H., Palma C.A., Gong Y., Hasch P., Elbing M., Mayor M., Reichert J., Barth J.V. (2018). Voltage-driven conformational switching with distinct Raman signature in a single-molecule junction. J. Am. Chem. Soc..

[bib10] Brooke R.J., Szumski D.S., Vezzoli A., Higgins S.J., Nichols R.J., Schwarzacher W. (2018). Dual control of molecular conductance through ph and potential in single-molecule devices. Nano Lett..

[bib11] Carlotti M., Soni S., Kumar S., Ai Y., Sauter E., Zharnikov M., Chiechi R.C. (2018). Two-terminal molecular memory through reversible switching of quantum interference features in tunneling junctions. Angew. Chem. Int. Ed..

[bib12] Chen X., Roemer M., Yuan L., Du W., Thompson D., del Barco E., Nijhuis C.A. (2017). Molecular diodes with rectification ratios exceeding 105 driven by electrostatic interactions. Nat. Nanotechnol..

[bib13] Cheng Z.L., Skouta R., Vazquez H., Widawsky J.R., Schneebeli S., Chen W., Hybertsen M.S., Breslow R., Venkataraman L. (2011). In situ formation of highly conducting covalent au-c contacts for single-molecule junctions. Nat. Nanotechnol..

[bib14] Choi S.H., Kim B., Frisbie C.D. (2008). Electrical resistance of long conjugated molecular wires. Science.

[bib15] Ciampi S., Darwish N., Aitken H.M., Diez-Perez I., Coote M.L. (2018). Harnessing electrostatic catalysis in single molecule, electrochemical and chemical systems: a rapidly growing experimental tool box. Chem. Soc. Rev..

[bib16] Darwish N., Diez-Perez I., Da Silva P., Tao N.J., Gooding J.J., Paddon-Row M.N. (2012). Observation of electrochemically controlled quantum interference in a single anthraquinone-based norbornylogous bridge molecule. Angew. Chem. Int. Ed..

[bib17] Dell E.J., Capozzi B., Xia J., Venkataraman L., Campos L.M. (2015). Molecular length dictates the nature of charge carriers in single-molecule junctions of oxidized oligothiophenes. Nat. Chem..

[bib18] Frisenda R., Janssen V.A., Grozema F.C., van der Zant H.S., Renaud N. (2016). Mechanically controlled quantum interference in individual pi-stacked dimers. Nat. Chem..

[bib19] Fujii S., Tada T., Komoto Y., Osuga T., Murase T., Fujita M., Kiguchi M. (2015). Rectifying electron-transport properties through stacks of aromatic molecules inserted into a self-assembled cage. J. Am. Chem. Soc..

[bib20] Garner M.H., Li H., Chen Y., Su T.A., Shangguan Z., Paley D.W., Liu T., Ng F., Li H., Xiao S. (2018). Comprehensive suppression of single-molecule conductance using destructive σ-interference. Nature.

[bib21] Gerhard L., Edelmann K., Homberg J., Valášek M., Bahoosh S.G., Lukas M., Pauly F., Mayor M., Wulfhekel W. (2017). An electrically actuated molecular toggle switch. Nat. Commun..

[bib22] Guedon C.M., Valkenier H., Markussen T., Thygesen K.S., Hummelen J.C., Van Der Molen S.J. (2012). Observation of quantum interference in molecular charge transport. Nat. Nanotechnol..

[bib23] Hines T., Díez-Pérez I., Nakamura H., Shimazaki T., Asai Y., Tao N. (2013). Controlling formation of single-molecule junctions by electrochemical reduction of diazonium terminal groups. J. Am. Chem. Soc..

[bib24] Hong W., Manrique D.Z., Moreno-Garcia P., Gulcur M., Mishchenko A., Lambert C.J., Bryce M.R., Wandlowski T. (2012). Single molecular conductance of tolanes: experimental and theoretical study on the junction evolution dependent on the anchoring group. J. Am. Chem. Soc..

[bib25] Huang X., Tang C., Li J., Chen L.C., Zheng J., Zhang P., Le J., Li R., Li X., Liu J. (2019). Electric field-induced selective catalysis of single-molecule reaction. Sci. Adv..

[bib26] Kocharova N., Ääritalo T., Leiro J., Kankare J., Lukkari J. (2007). Aqueous dispersion, surface thiolation, and direct self-assembly of carbon nanotubes on gold. Langmuir.

[bib27] Koren E., Leven I., Lortscher E., Knoll A., Hod O., Duerig U. (2016). Coherent commensurate electronic states at the interface between misoriented graphene layers. Nat. Nanotechnol..

[bib28] Lambert C.J. (2015). Basic concepts of quantum interference and electron transport in single-molecule electronics. Chem. Soc. Rev..

[bib29] Leary E., La Rosa A., González M.T., Rubio-Bollinger G., Agraït N., Martín N. (2015). Incorporating single molecules into electrical circuits. The role of the chemical anchoring group. Chem. Soc. Rev..

[bib30] Li L.W., Lo W.Y., Cai Z.X., Zhang N., Yu L.P. (2016). Proton-triggered switch based on a molecular transistor with edge-on gate. Chem. Sci..

[bib31] Li R., Lu Z., Cai Y., Jiang F., Tang C., Chen Z., Zheng J., Pi J., Zhang R., Liu J. (2017). Switching of charge transport pathways via delocalization changes in single-molecule metallacycles junctions. J. Am. Chem. Soc..

[bib32] Li Y., Buerkle M., Li G., Rostamian A., Wang H., Wang Z., Bowler D.R., Miyazaki T., Xiang L., Asai Y. (2019). Gate controlling of quantum interference and direct observation of anti-resonances in single molecule charge transport. Nat. Mater..

[bib33] Liu X., Sangtarash S., Reber D., Zhang D., Sadeghi H., Shi J., Xiao Z.Y., Hong W., Lambert C.J., Liu S.X. (2017). Gating of quantum interference in molecular junctions by heteroatom substitution. Angew. Chem. Int. Ed..

[bib34] Liu J., Huang X., Wang F., Hong W. (2019). Quantum interference effects in charge transport through single-molecule junctions: detection, manipulation, and application. Acc. Chem. Res..

[bib35] Lörtscher E., Ciszek J.W., Tour J., Riel H.J.S. (2006). Reversible and controllable switching of a single-molecule junction. Small.

[bib36] Mayor M., Weber H.B., Reichert J., Elbing M., von Hänisch C., Beckmann D., Fischer M. (2003). Electric current through a molecular rod—relevance of the position of the anchor groups. Angew. Chem. Int. Ed..

[bib37] Meded V., Bagrets A., Arnold A., Evers F.J.S. (2009). Molecular switch controlled by pulsed bias voltages. Small.

[bib38] Meisner J.S., Ahn S., Aradhya S.V., Krikorian M., Parameswaran R., Steigerwald M., Venkataraman L., Nuckolls C. (2012). Importance of direct metal-pi coupling in electronic transport through conjugated single-molecule junctions. J. Am. Chem. Soc..

[bib39] Meng L., Xin N., Hu C., Wang J., Gui B., Shi J., Wang C., Shen C., Zhang G., Guo H. (2019). Side-group chemical gating via reversible optical and electric control in a single molecule transistor. Nat. Commun..

[bib40] Miguel D., Alvarez de Cienfuegos L., Martín-Lasanta A., Morcillo S.P., Zotti L.A., Leary E., Bürkle M., Asai Y., Jurado R., Cárdenas D.J. (2015). Toward multiple conductance pathways with heterocycle-based oligo (phenyleneethynylene) derivatives. J. Am. Chem. Soc..

[bib41] Moth-Poulsen K., Bjørnholm T. (2009). Molecular electronics with single molecules in solid-state devices. Nat. Nanotechnol..

[bib42] Olavarria-Contreras I.J., Etcheverry-Berrios A., Qian W.J., Gutierrez-Ceron C., Campos-Olguin A., Sanudo E.C., Dulic D., Ruiz E., Aliaga-Alcalde N., Soler M. (2018). Electric-field induced bistability in single-molecule conductance measurements for boron coordinated curcuminoid compounds. Chem. Sci..

[bib43] Quek S.Y., Kamenetska M., Steigerwald M.L., Choi H.J., Louie S.G., Hybertsen M.S., Neaton J.B., Venkataraman L. (2009). Mechanically controlled binary conductance switching of a single-molecule junction. Nat. Nanotechnol..

[bib44] Ratner M. (2013). A brief history of molecular electronics. Nat. Nanotechnol..

[bib45] Shaik S., Mandal D., Ramanan R. (2016). Oriented electric fields as future smart reagents in chemistry. Nat. Chem..

[bib46] Shaik S., Ramanan R., Danovich D., Mandal D. (2018). Structure and reactivity/selectivity control by oriented-external electric fields. Chem. Soc. Rev..

[bib47] Solomon G.C., Herrmann C., Hansen T., Mujica V., Ratner M.A. (2010). Exploring local currents in molecular junctions. Nat. Chem..

[bib48] Su T.A., Neupane M., Steigerwald M.L., Venkataraman L., Nuckolls C. (2016). Chemical principles of single-molecule electronics. Nat. Rev. Mater..

[bib49] Tang C., Chen L., Zhang L., Chen Z., Li G., Yan Z., Lin L., Liu J., Huang L., Ye Y. (2019). Multicenter-bond-based quantum interference in charge transport through single-molecule carborane junctions. Angew. Chem. Int. Ed..

[bib50] Thompson D., Nijhuis C.A. (2016). Even the odd numbers help: failure modes of sam-based tunnel junctions probed via odd-even effects revealed in synchrotrons and supercomputers. Acc. Chem. Res..

[bib51] Vergeer F.W., Chen X.D., Lafolet F., De Cola L., Fuchs H., Chi L.F. (2006). Ultrathin luminescent films of rigid dinuclear ruthenium(ii) trisbipyridine complexes. Adv. Funct. Mater..

[bib52] Wang Z., Danovich D., Ramanan R., Shaik S. (2018). Oriented-external electric fields create absolute enantioselectivity in diels–alder reactions: importance of the molecular dipole moment. J. Am. Chem. Soc..

[bib53] Xiang D., Wang X., Jia C., Lee T., Guo X. (2016). Molecular-scale electronics: from concept to function. Chem. Rev..

[bib54] Xiang L.M., Hines T., Palma J.L., Lu X.F., Mujica V., Ratner M.A., Zhou G., Tao N.J. (2016). Non-exponential length dependence of conductance in iodide terminated oligothiophene single-molecule tunneling junctions. J. Am. Chem. Soc..

[bib55] Yanson A.I., Bollinger G.R., van den Brom H.E., Agrait N., van Ruitenbeek J.M. (1998). Formation and manipulation of a metallic wire of single gold atoms. Nature.

[bib56] Yoshizawa K., Tada T., Staykov A. (2008). Orbital views of the electron transport in molecular devices. J. Am. Chem. Soc..

[bib57] Zhang L., Laborda E., Darwish N., Noble B.B., Tyrell J.H., Pluczyk S., Le Brun A.P., Wallace G.G., Gonzalez J., Coote M.L. (2018). Electrochemical and electrostatic cleavage of alkoxyamines. J. Am. Chem. Soc..

