# Supplementary material for: Electric-Field-Induced Connectivity Switching in Single-Molecule Junctions
Source: iScience. 2019 Dec 14;23(1):100770. doi: 10.1016/j.isci.2019.100770 (PMC6970166; doi:10.1016/j.isci.2019.100770)
Supplement: Document S1. Transparent Methods and Figures S1–S28 [file mmc1.pdf]

ISCI, Volume *23*

Supplemental Information

Electric-Field-Induced Connectivity Switching in Single-Molecule Junctions

Chun Tang, Jueting Zheng, Yiling Ye, Junyang Liu, Lijue Chen, Zhewei Yan, Zhixin Chen, Lichuan Chen, Xiaoyan Huang, Jie Bai, Zhaobin Chen, Jia Shi, Haiping Xia, and Wenjing Hong

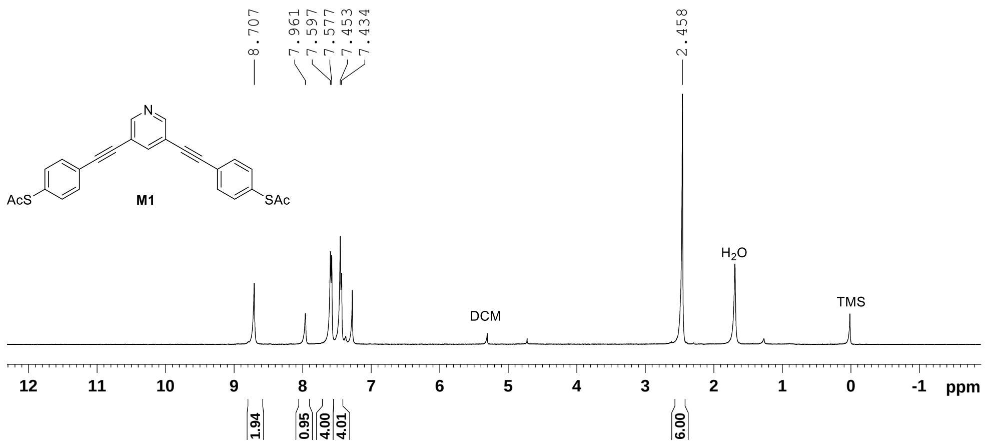

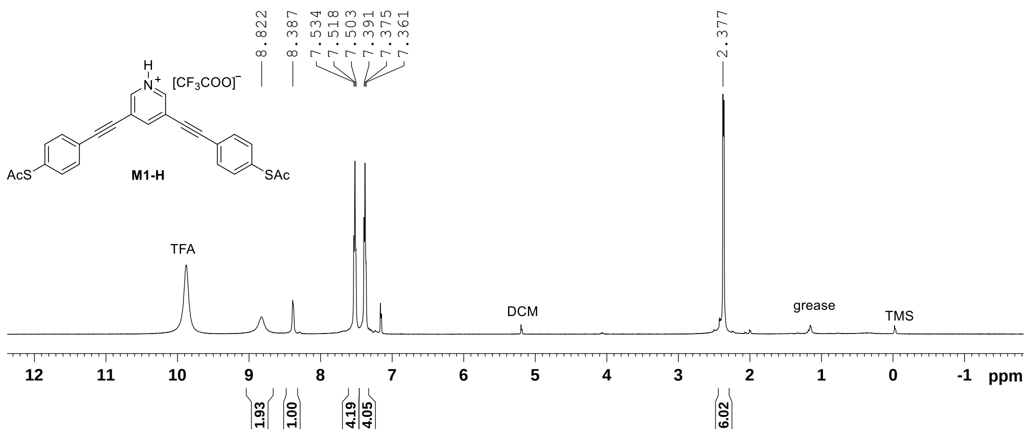

Figure S1. ^{1}H NMR ($CDCl_3$, 500 MHz) spectrum of compound **M1-H** in comparison with **M1**. Related to Figure 2.

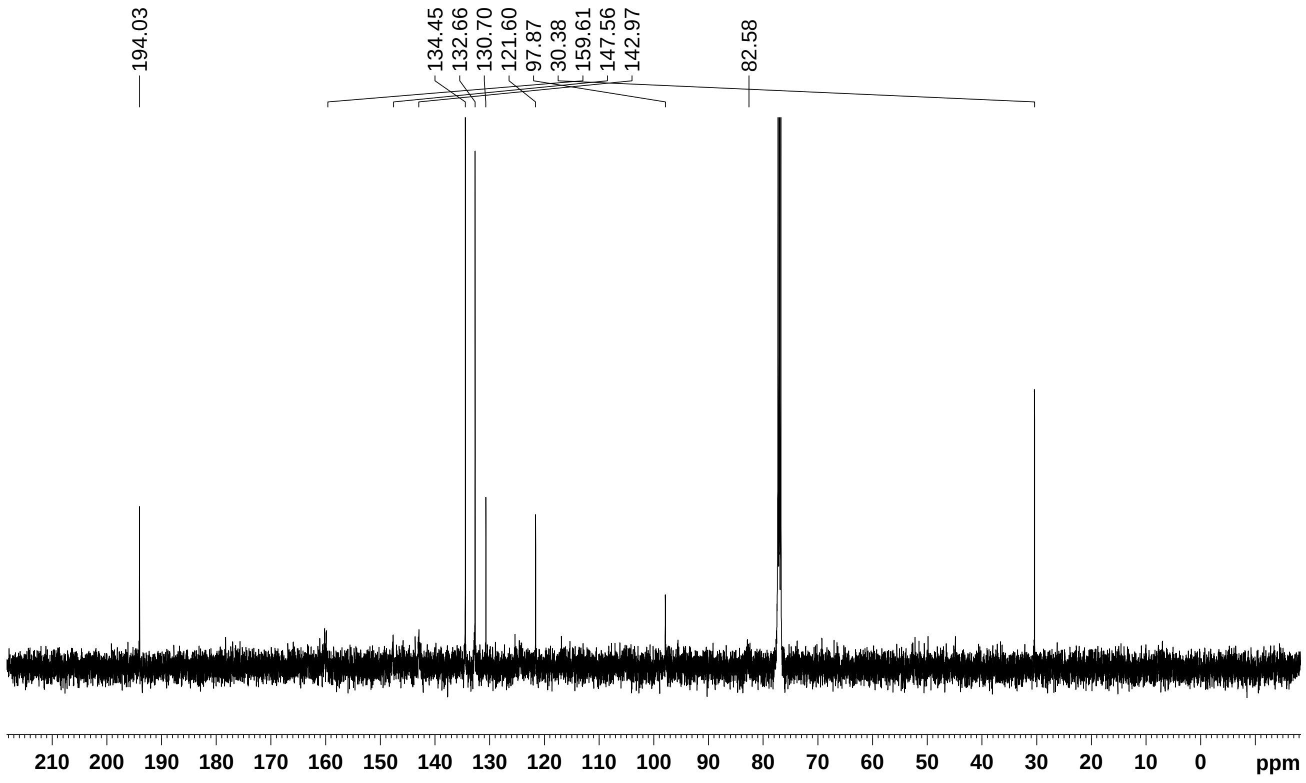

Figure S2. ^{13}C NMR ($CDCl_3$, 125 MHz) spectrum of compound **M1-H**. Related to Figure 2.

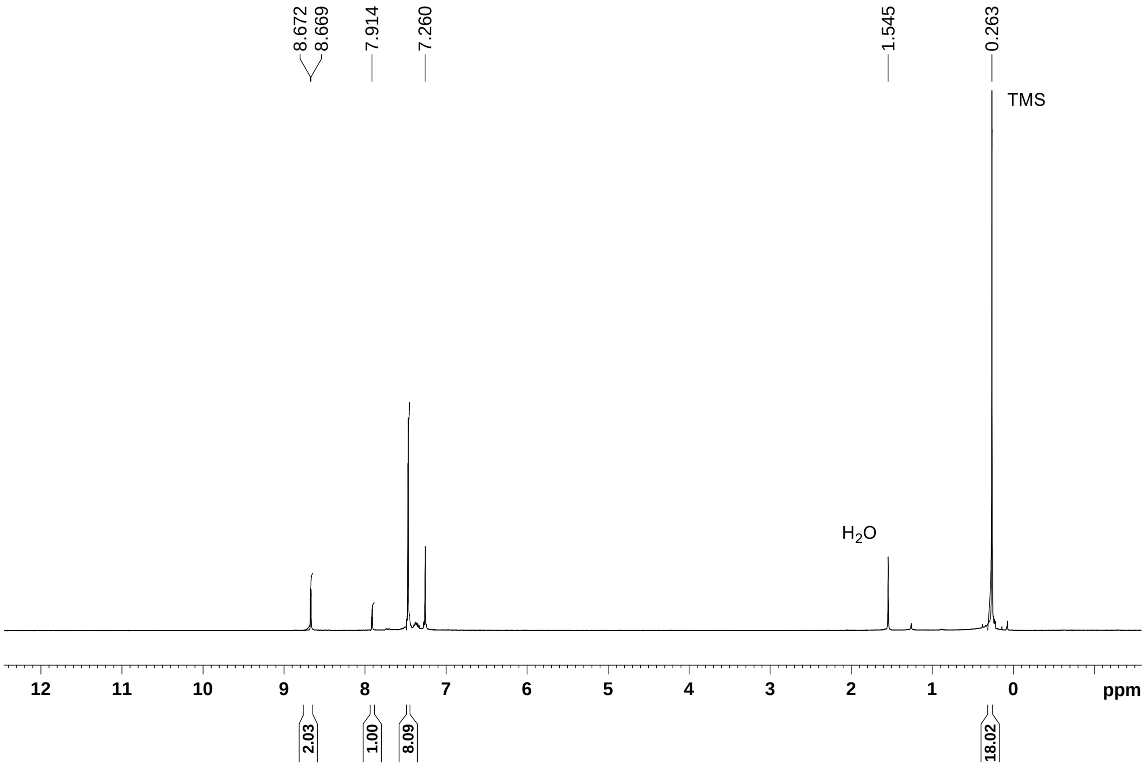

Figure S3. ^{1}H NMR ($CDCl_3$, 500 MHz) spectrum of compound **2**. Related to Figure 2.

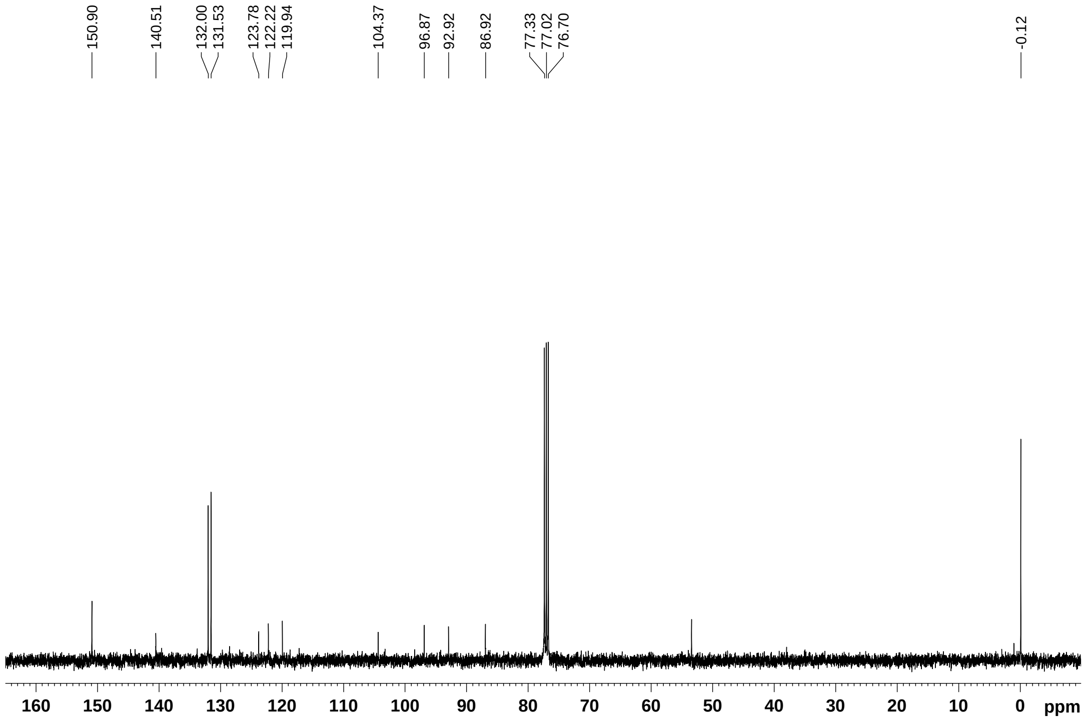

Figure S4. ^{13}C NMR ($CDCl_3$, 125 MHz) spectrum of compound **2**. Related to Figure 2.

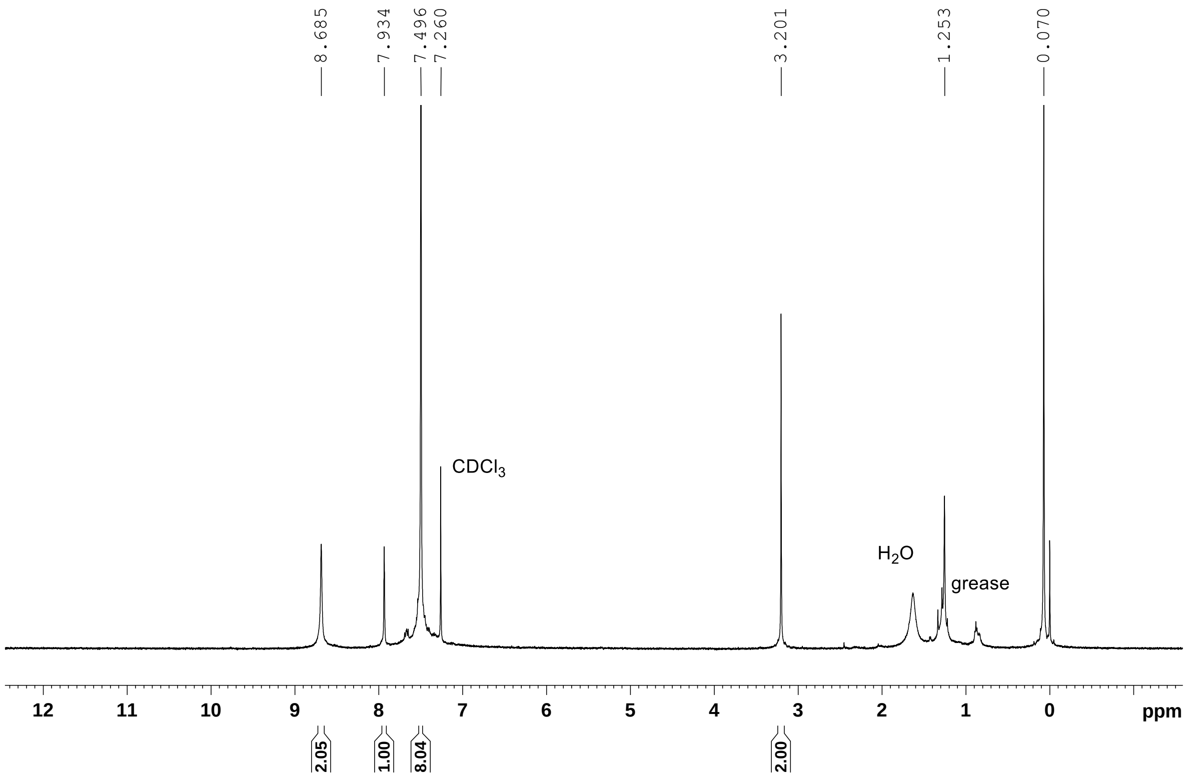

Figure S5. ^{1}H NMR ($CDCl_3$, 500 MHz) spectrum of compound **3**. Related to Figure 2.

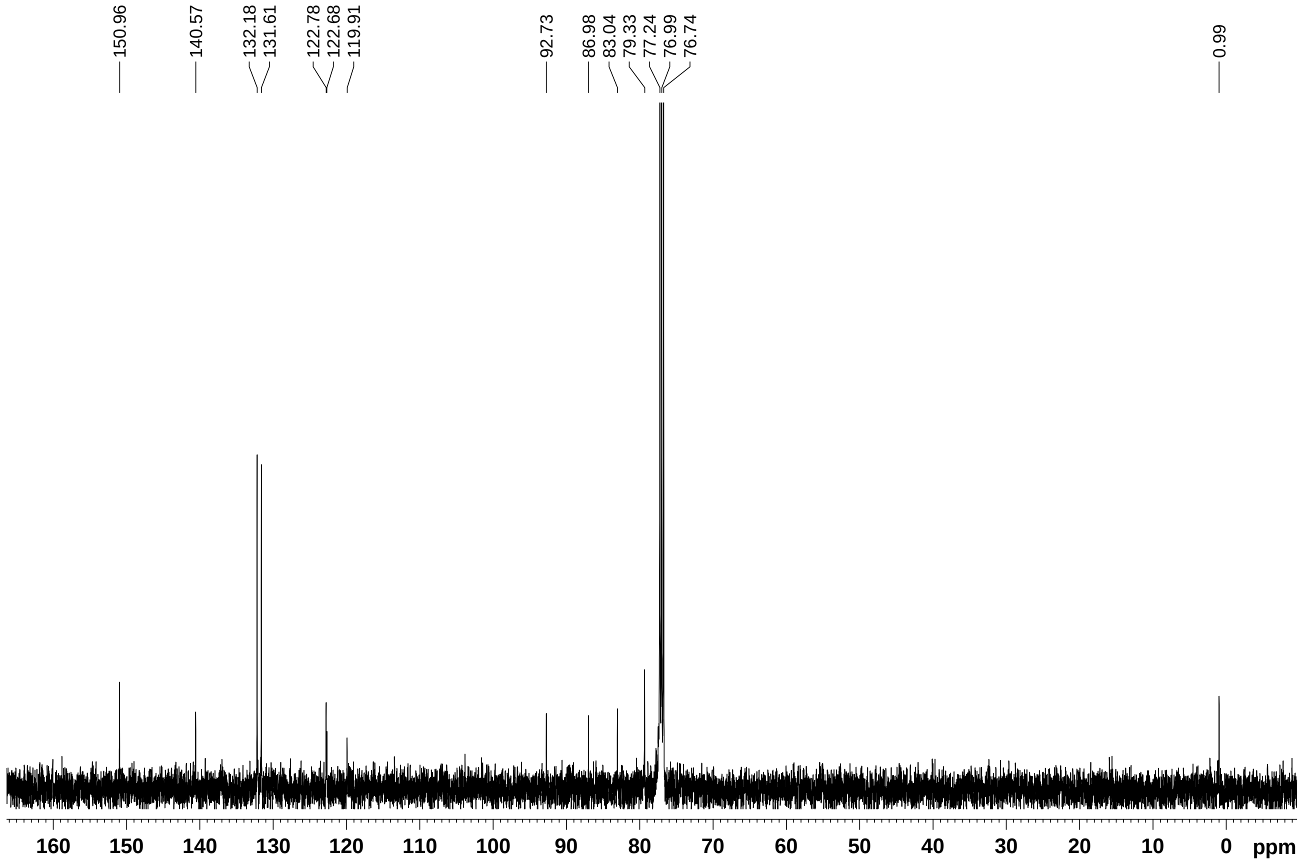

Figure S6. ^{13}C NMR ($CDCl_3$, 125 MHz) spectrum of compound **3**. Related to Figure 2.

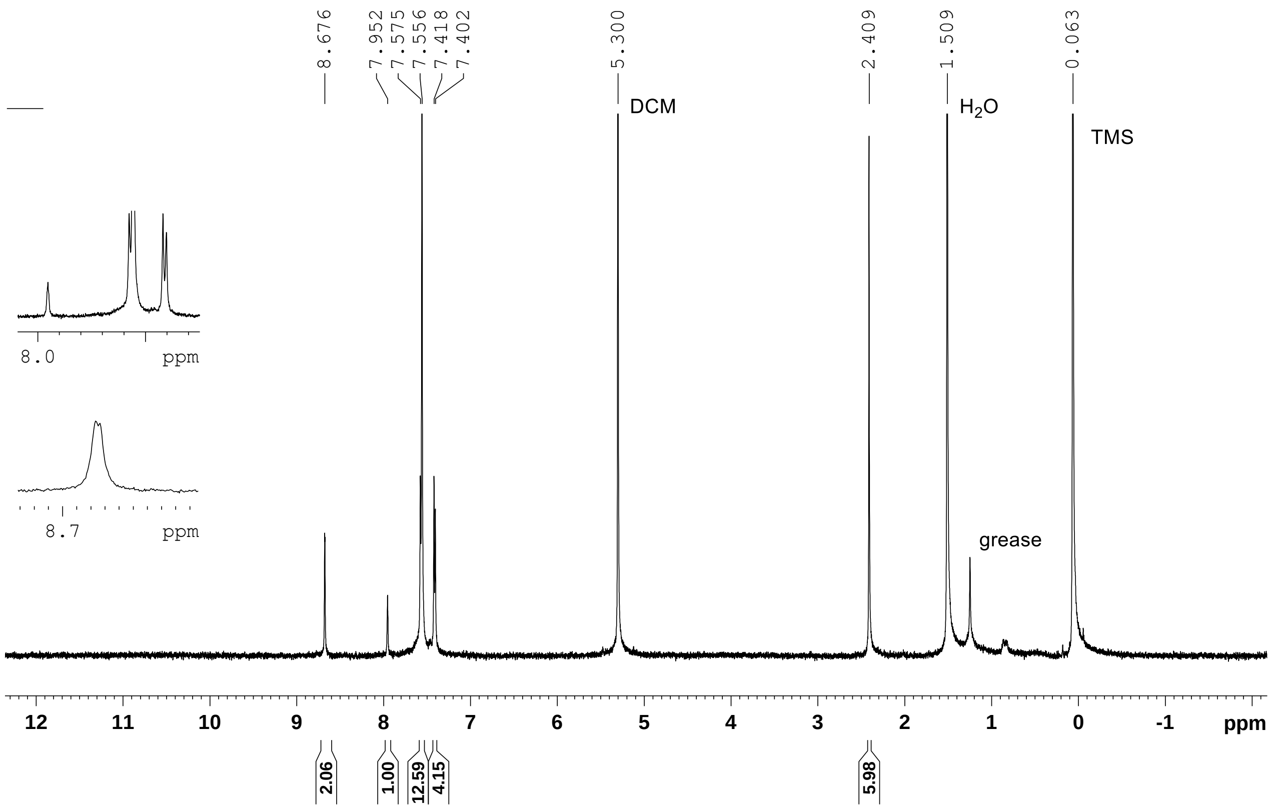

Figure S7. ^{1}H NMR (CD_2Cl_2, 500 MHz) spectrum of compound **M1L**. Related to Figure 2.

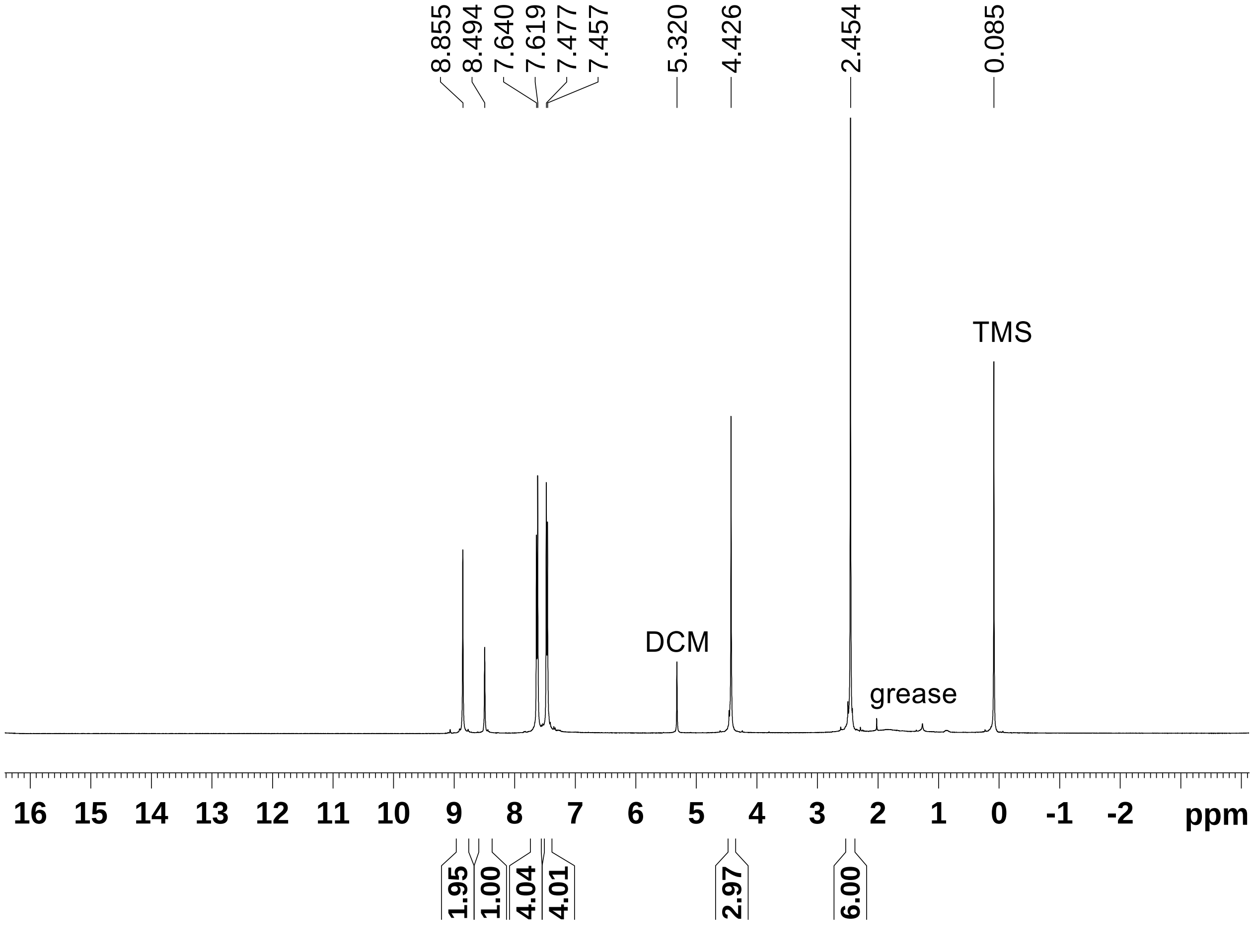

Figure S8. ^{1}H NMR (CD_2Cl_2, 500 MHz) spectrum of compound **M1-Me**. Related to Figure 2.

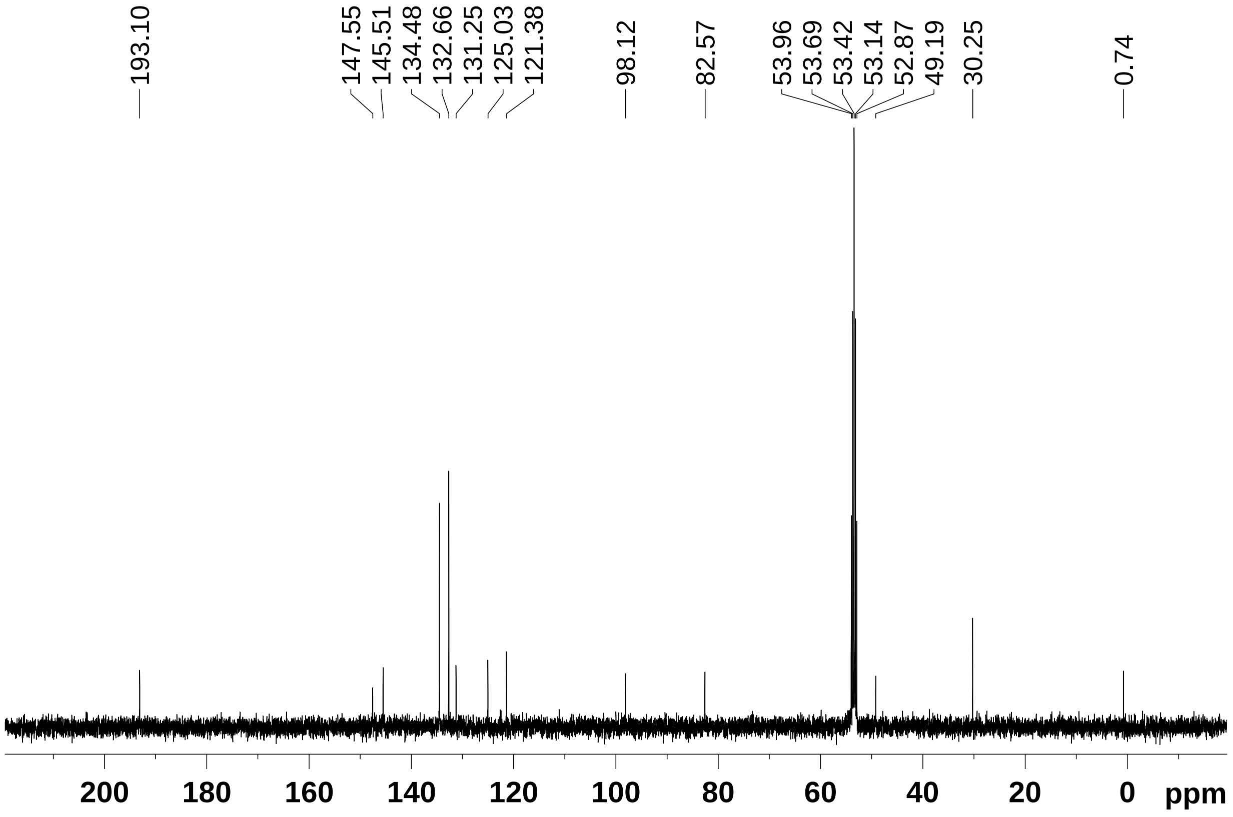

Figure S9. ^{13}C NMR (CD_2Cl_2, 125 MHz) spectrum of compound **M1-Me**. Related to Figure 2.

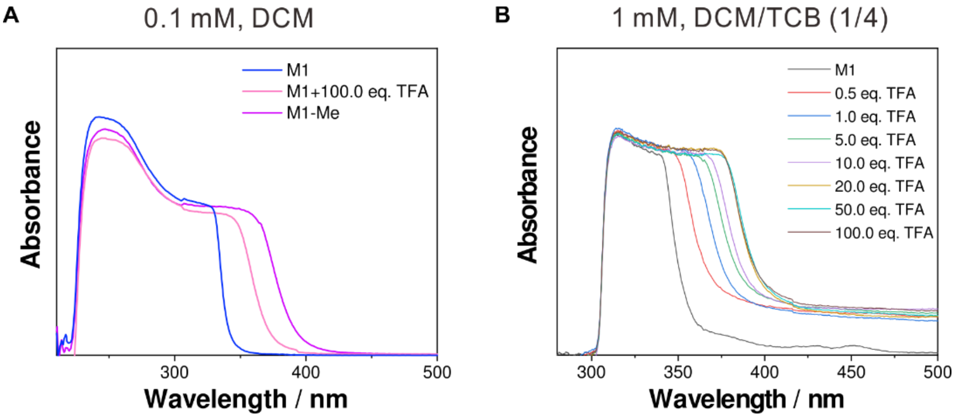

Figure S10. (**A**) UV-Vis spectra of methylated pyridiniums **M1-H** and **M1-Me** and their pyridine parent **M1** with 0.1 mM concentration in the solvent of DCM. The solution of **M1-H** is formed with 0.1 mM **M1** and 100 eq. TFA. (**B**) UV-Vis spectra of 1 mM **M1** in solvent mixture of DCM/TCB (1/4) with different equivalent of TFA added. Related to Figure 2.

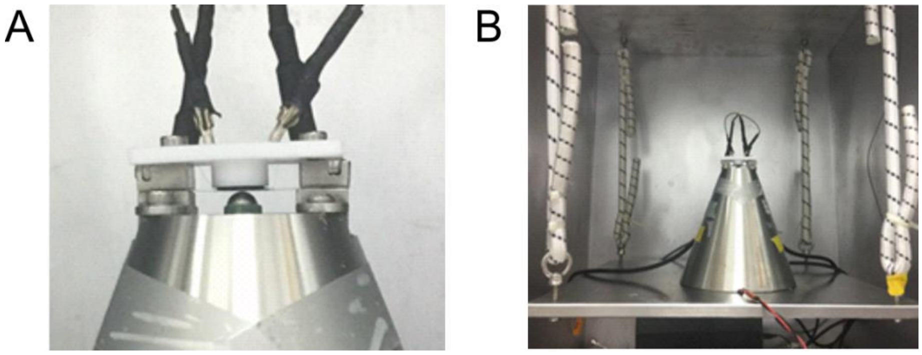

Figure S11. (**A**) Side view of the MCBJ setup. (**B**) Overview of the MCBJ setup with a suspension to damp vibration. Related to Figure 2.

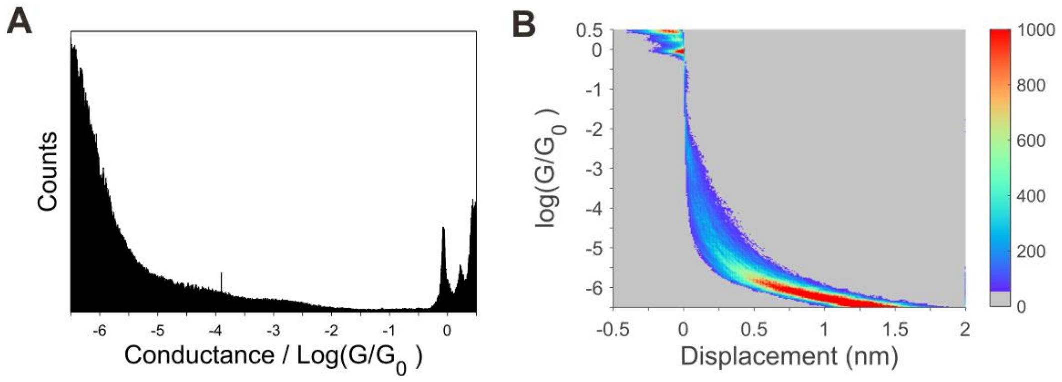

Figure S12. All data-point one-dimensional (**A**) and two-dimensional(**B**) conductance histograms constructed from two thousand MCBJ traces for the solvent DCM/TCB (*v*/*v*,1/4). The above measurements were perform at room temperature with 0.10 V bias applied. Related to Figure 2.

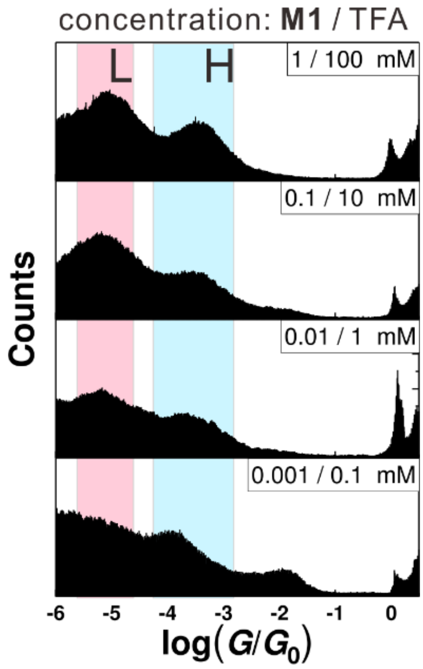

Figure S13. The one-dimensional conductance histograms by varying the concentrations between **M1** and TFA (with the ratio of 1/100). Related to Figure 2.

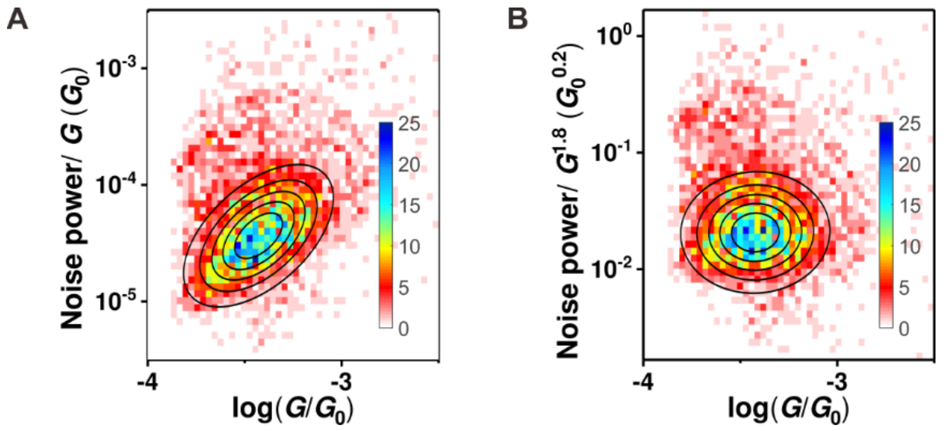

Figure S14. Two-dimensional histogram of normalized flicker noise power versus average conductance for the high-conductance junctions of **M1-H** normalized by $G^{1.0}$ (**A**), and normalized by $G^{1.8}$ (**B**). Related to Figure 2.

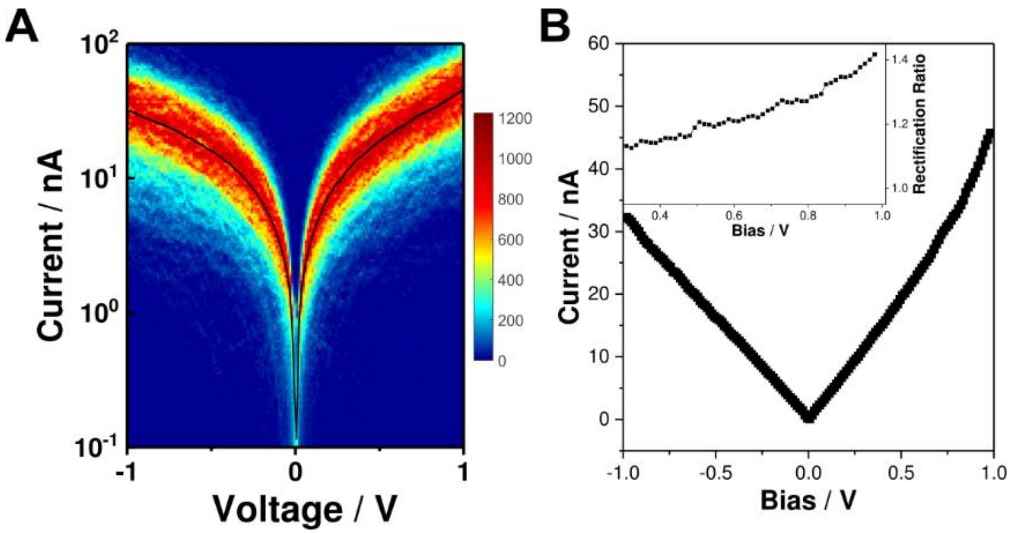

Figure S15. (**A**) The two-dimensional *I*/*V* histogram for **M1-H** constructed from about 2000 traces. The I/V histogram is fitted by Gaussian distribution shown by the black solid line. (**B**) The linear histogram for the fitted I/V character with the rectification ratio shown inset. Related to Figure 2.

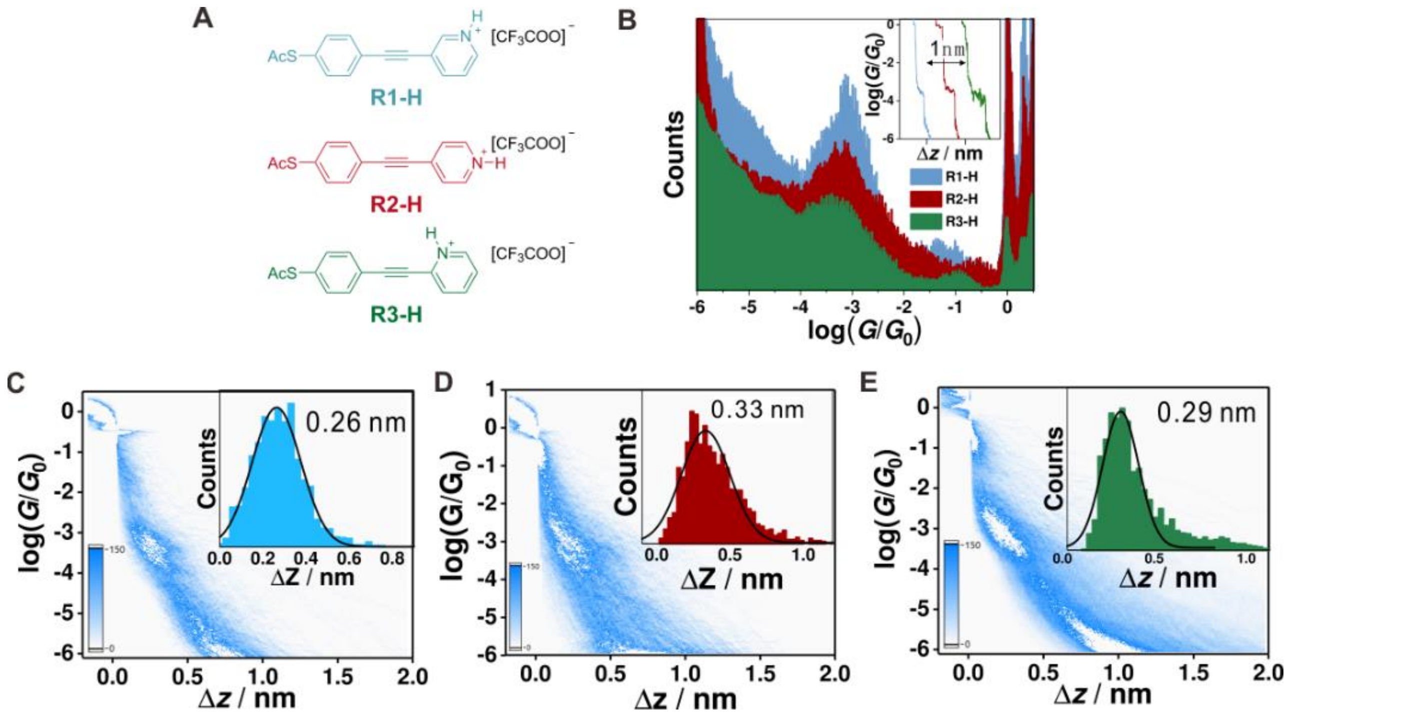

Figure S16. (**A**) Molecular structures of reference molecules. (**B**) All data-point one-dimensional conductance histograms constructed from about two thousand conductance traces. (**C**-**E**) Two-dimensional conductance histograms of **R1-H** (**C**), **R2-H** (**D**) and **R3-H** (**E**) with stretching distance Δz distributions shown inset. Related to Figure 2.

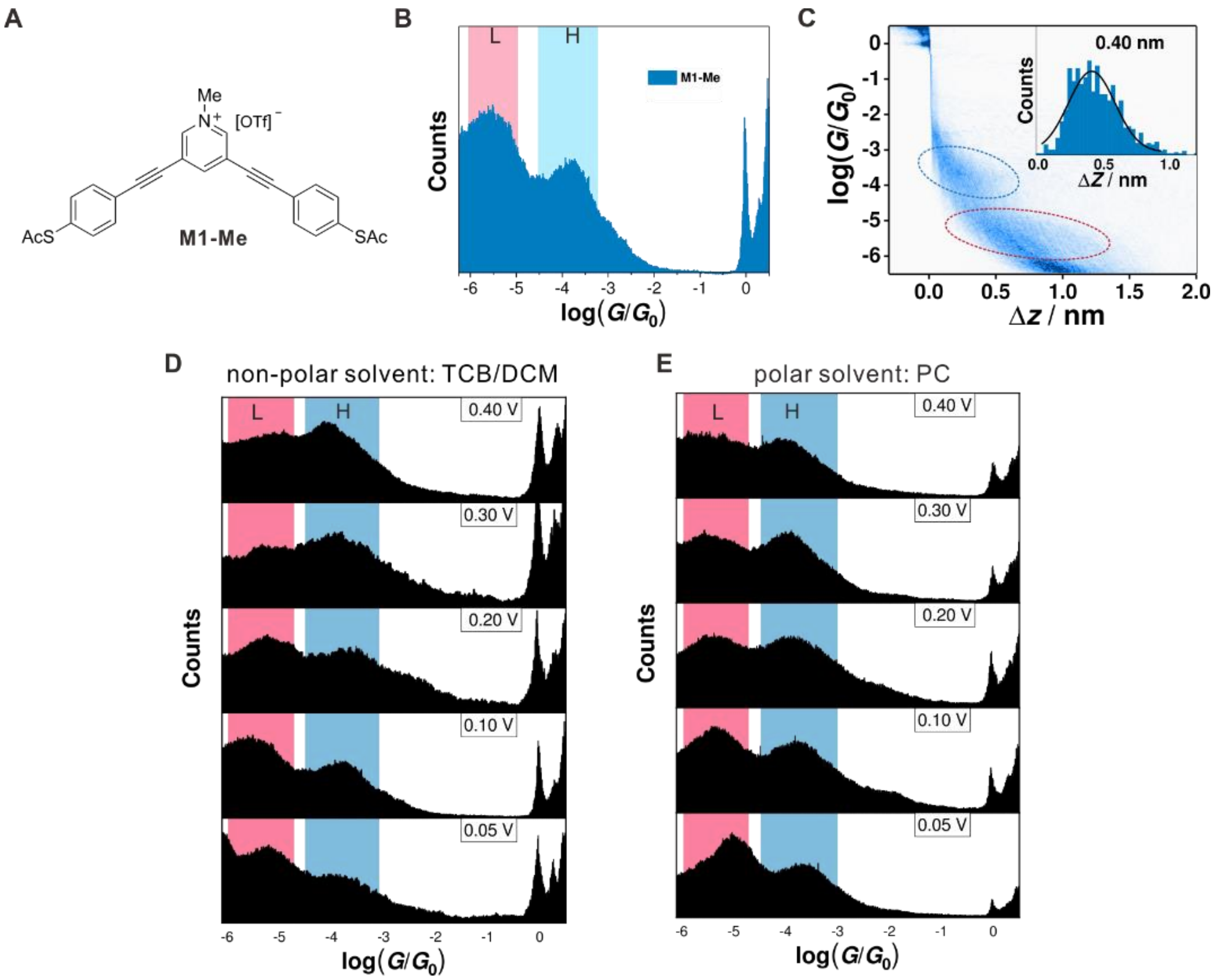

Figure S17. (**A**) Molecular structures of **M1-Me**. (**B**) All data-point one-dimensional conductance histograms constructed from about two thousand conductance traces. (**C**) Two-dimensional conductance histograms of **M1-Me** with stretching distance Δ*z* distributions shown inset. (**D**) One-dimensional conductance histograms of **M1-Me** with a different bias applied, in the solvent TCB/DCM mixture (*v*/*v*, 4/1). (**E**) One-dimensional conductance histograms of **M1-Me** with a different bias applied, in the solvent of propylene carbonate (PC). Related to Figure 2 and Figure 3.

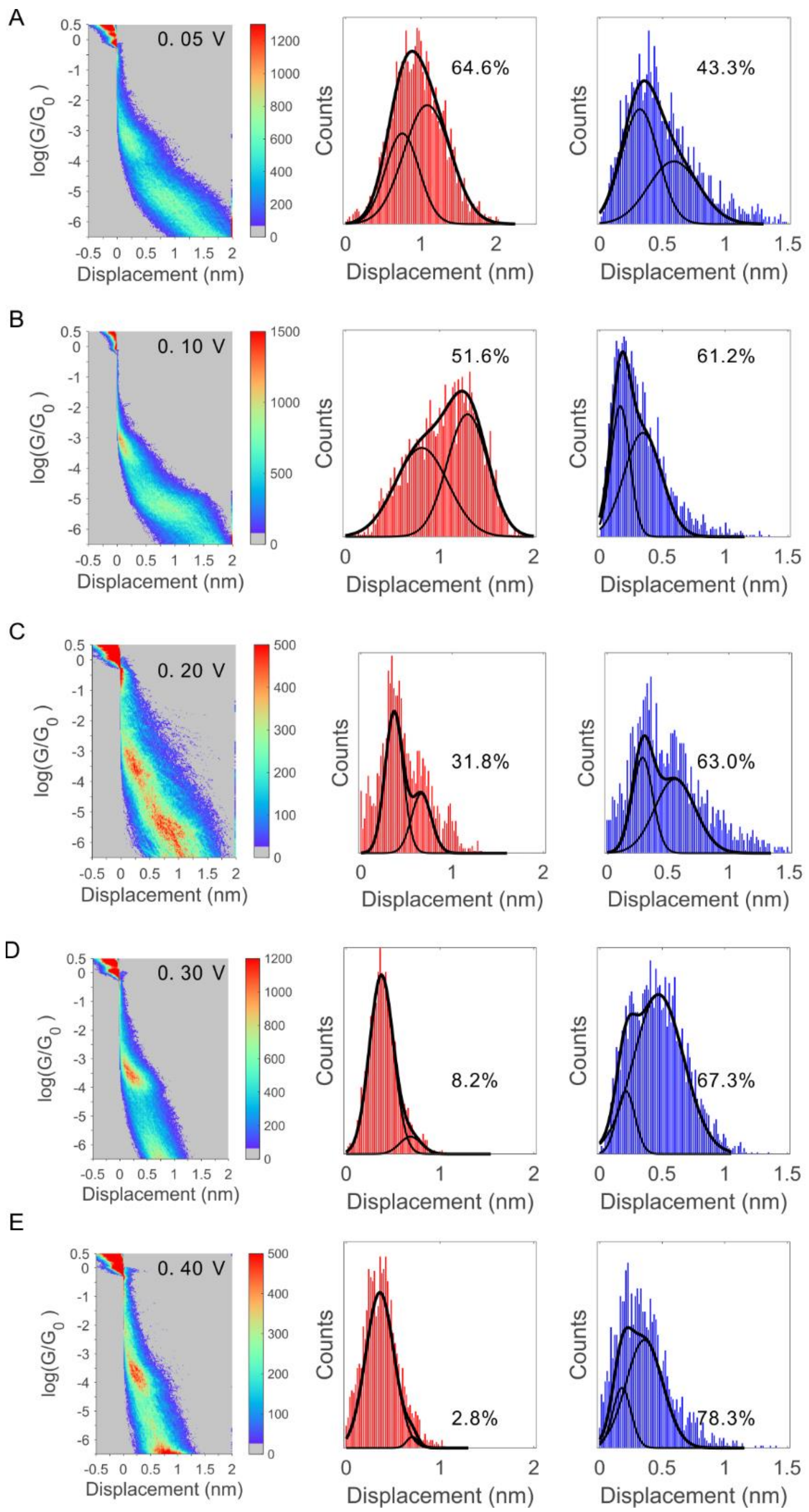

Figure S18. 2D conductance histogram of **M1-H** in the bias of 0.05 (a), 0.10 (b), 0.20 (c), 0.30 (d), and 0.40 V (e) respectively. Junction formation probability analyzing for the low conductance regions are shown in the middle panels, while that for the low conductance regions are shown in the right panels. Related to Figure 3A.

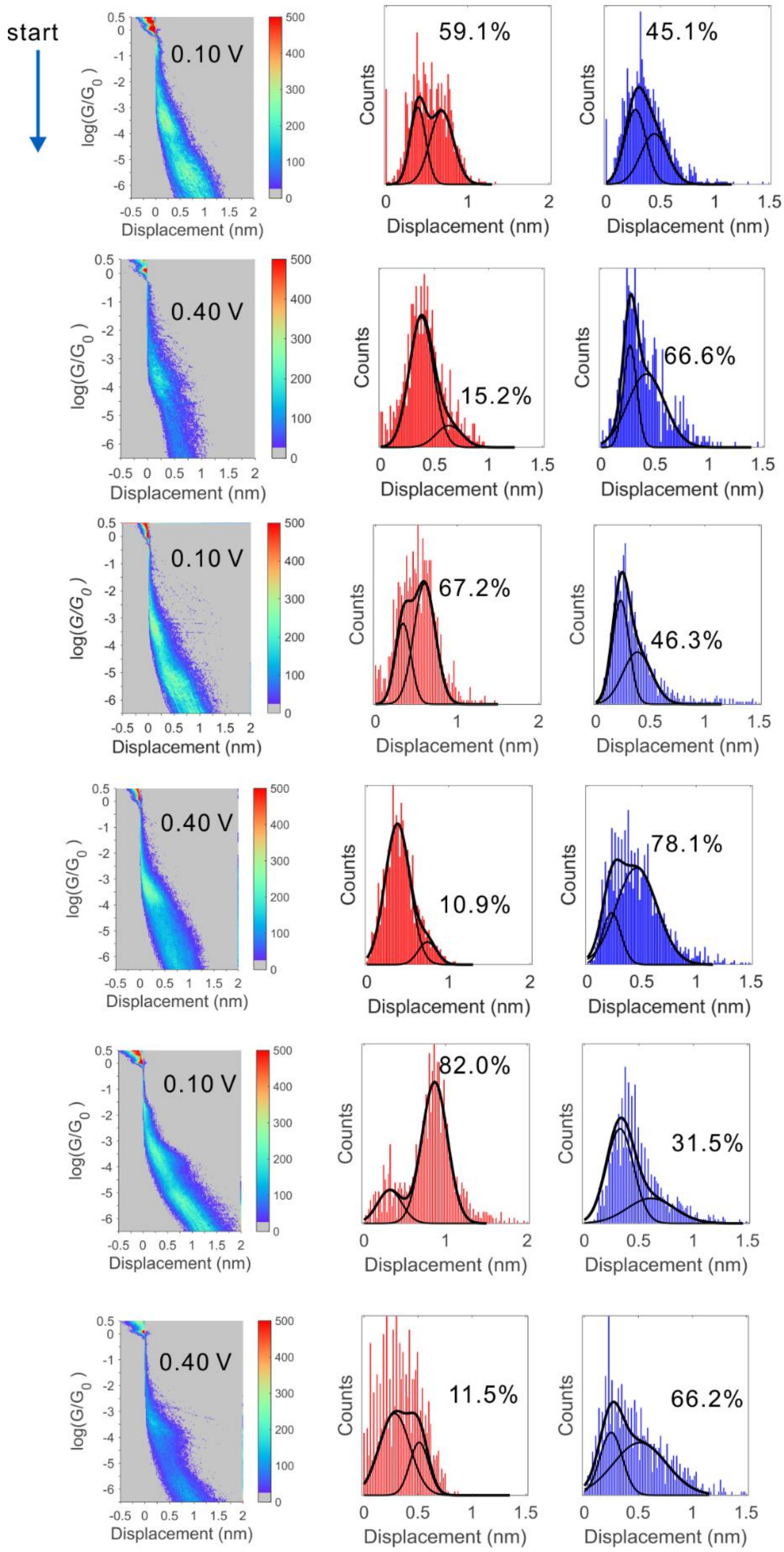

Figure S19. 2D conductance histogram (left panel) of **M1-H**. Junction formation probability analyzing for the low- and high-conductance regions are shown in the middle and right panels. Related to Figure 3D.

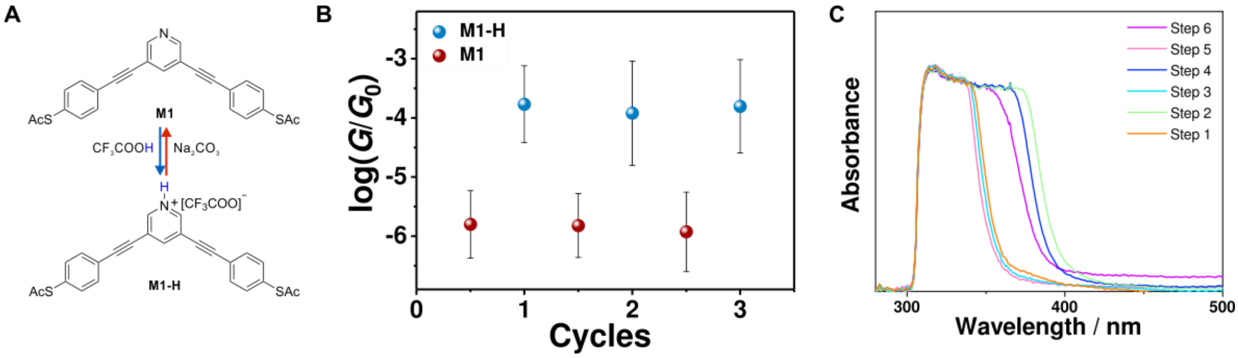

Figure S20. (**A**) The structures of **M1-H** and **M1**, which show reversible switching with base or acid added. (**B**) Reversible switching of molecular conductance between **M1-H** and **M1** with acid or base treatment successively. The MCBJ experiments were performed under a solvent mixture (DCM/TCB = 1/4) with 0.10 V bias applied. The reversibility between **M1** and **M1-H** was accomplished as mentioned above. (**C**) UV-Vis spectra of the layer of solvent mixture (DCM/TCB = 1/4) in each cycle step. Related to Figure 2.

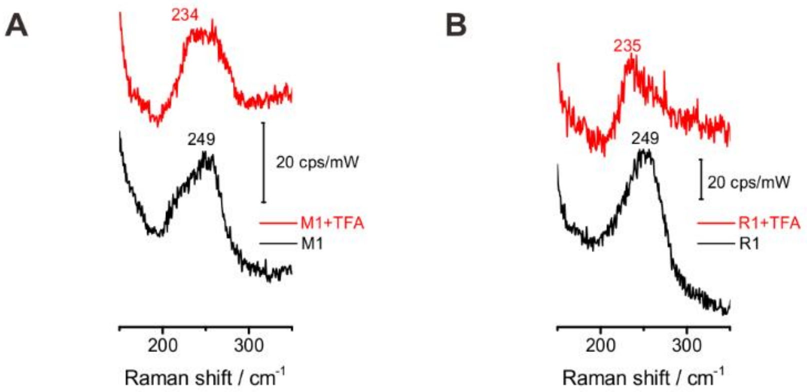

Figure S21. Surface enhance Raman spectra. **M1**/ **M1-H** (**A**) and **R1**/ **R1-H** (**B**) on gold nanoparticles. Related to Figure 4E.

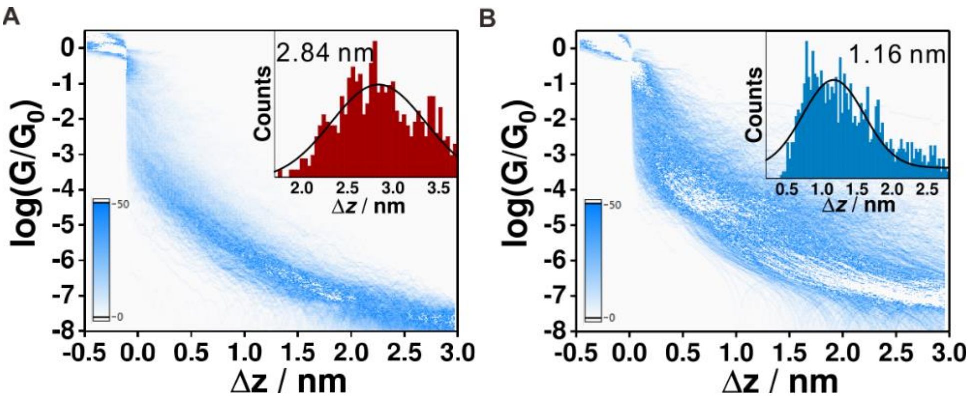

Figure S22 Two-dimensional conductance histograms of **M1L** (**A**) and **M1L-H** (**B**) with stretching distance Δz distributions shown inset. It is worth noting that the most probable conductance for the low-conductance junctions of **M1L** is lower than $10^{-7.1}$ G_0, which is below the detecting limit of our devices. Only 15% single traces of **M1L** show molecular platitudes, leading to the molecular peak centered at $10^{-7.1}$ G_0, which is the distribution of the high value area. We also tried to measure the low-conductance junction of **M1L-H**, but the background noise ($10^{-7.0}$ G_0) is alway higher that the measurement in neutral state **M1L**, thus we think the low-conductance junctions of **M1L-H** should below $10^{-7.0}$ G_0. We found that the molecules similar to **M1L** with *para*-connectivity has a conductance centered at $10^{-7.0}$ G_0, which means the most probable conductance for corresponding molecules in *meta*-connectivity should below $10^{-8.0}$ G_0. We actually underestimate the conductance difference between the two connectivities of **M1L-H**, which should have far more conductance difference than 400 times. Related to Figure 2.

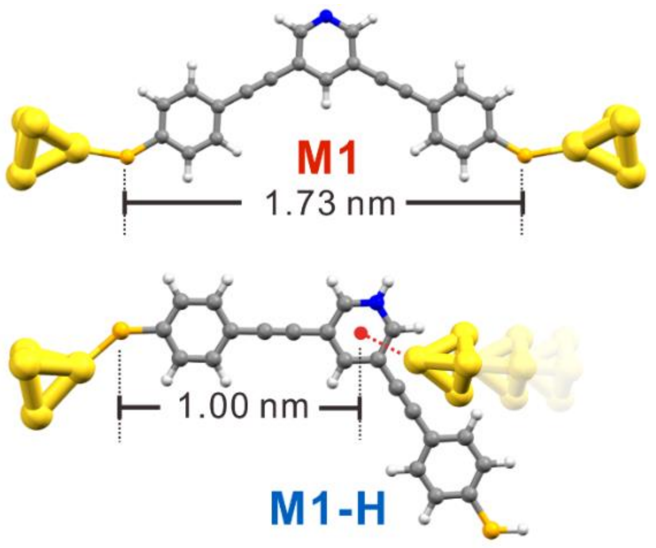

Figure S23. The end-to-end connectivity for **M1** (top), and the in-backbone connectivity for **M1-H** (bottom), with theoretical lengths 1.73 and 1.00 nm respectively. Related to Figure 2.

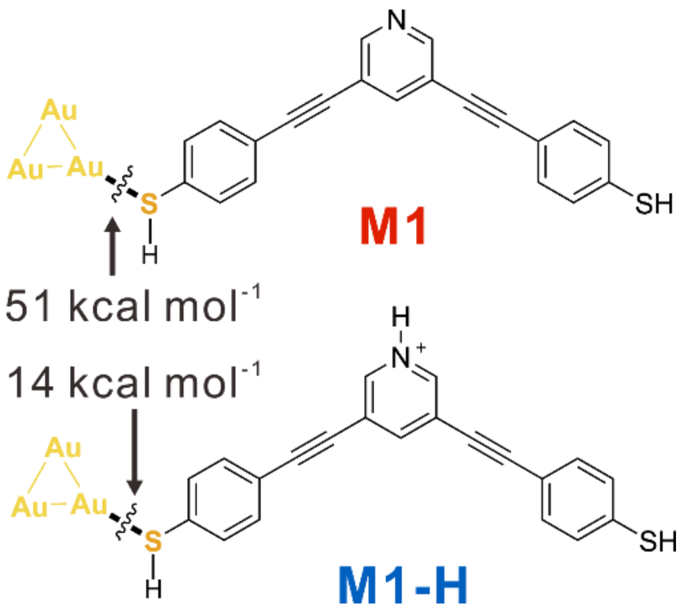

Figure S24. The Au←SH coordination bond formation energy of **M1** and **M1-H.** Related to Figure 4E.

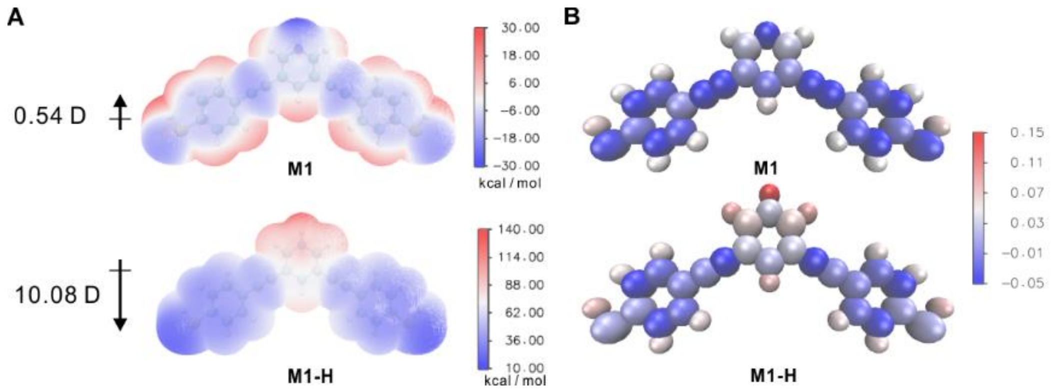

Figure S25. (**A**) The electrostatic potential distributions of **M1** and **M1-H**, with their dipole moments shown by the arrows. (**B**) The atomic charge distribution of **M1** and **M1-H**. Related to Figure 4.

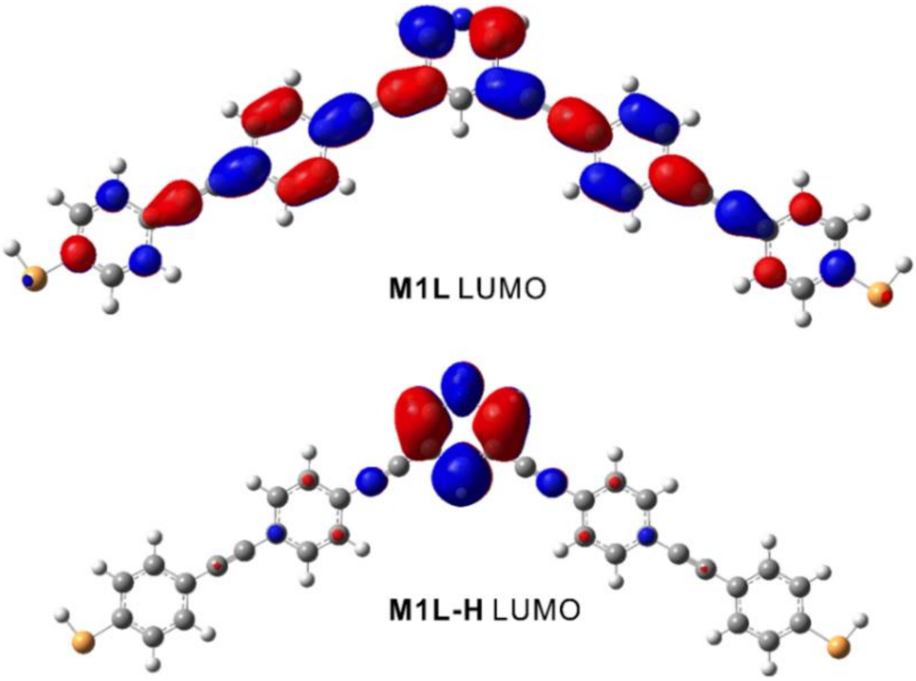

Figure S26. The upper panel is the LUMO of **M1L** and the bottom panel is the LUMO of **M1L-H**. Related to Figure 4.

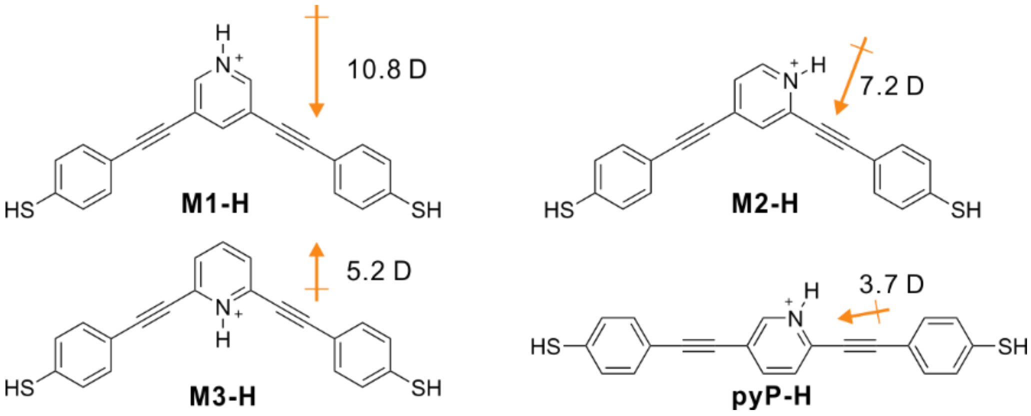

Figure S27. The strength and direction of the dipole moments of the protonated pyridiniums with nitrogen in different positions. Related to Figure 4.

Transparent Methods

Section 1. Synthesis and Characterization

General Information.

Commercially available reagents and solvents obtained from chemical suppliers were used without further purification unless otherwise noted. Nuclear magnetic resonance (NMR) spectroscopic experiments were performed on Bruker AV–500 spectrometers (400 MHz). ^{1}H NMR spectra were recorded at 400 MHz and chemical shifts are reported in ppm using residual deuterated solvent peak as reference ($CDCl_3$: δ 7.26, CD_2Cl_2: δ 5.30). The following abbreviations were used to explain the multiplicities: s = singlet, d = doublet, t = triplet, q = quartet, m = multiplet, br = broad. ^{13}C NMR spectra were recorded at 100 MHz using broadband proton decoupling and chemical shifts are reported in ppm relative to residual deuterated solvent peak ($CDCl_3$: δ 77.00). High-resolution mass spectra (HRMS) experiments were recorded on a Bruker En Apex Ultra 7.0T Fourier Transform Mass Spectrometer.

Preparation and characterization

Compound **M1**, **R1**, **R2,** and **R3** were synthesized according to the previous results(Liu et al., 2017b). Compound **M1L** was synthesized with similar protocol.

To characterized the NMR of **M1-H** and reduce the interference of TFA, we added 5 eq. TFA to the solution ($CDCl_3$) of **M1**, leading to the formation of **M1-H** in-situ.

Characterization of compound **M1-H**: ^{1}H NMR (400 MHz, $CDCl_3$): δ = 8.82 (br, 2H), 8.39 (s, 1H), 7.52 (t, J = 8.52 Hz, 4H), 7.37 (t, J = 8.50 Hz, 4H), and 2.37 (s, 6H). ^{13}C NMR (100 MHz, CD_2Cl_2): δ = 194.05, 159.61, 147.56, 142.97, 134.45, 132.66, 130.70, 121.60, 97.87, 82.57 and 30.38. HRMS (ESI): *m/z* calcd for $[C_{25}H_{18}NO_2S_2]^+$, 428.0773 ; found, 428.0735.

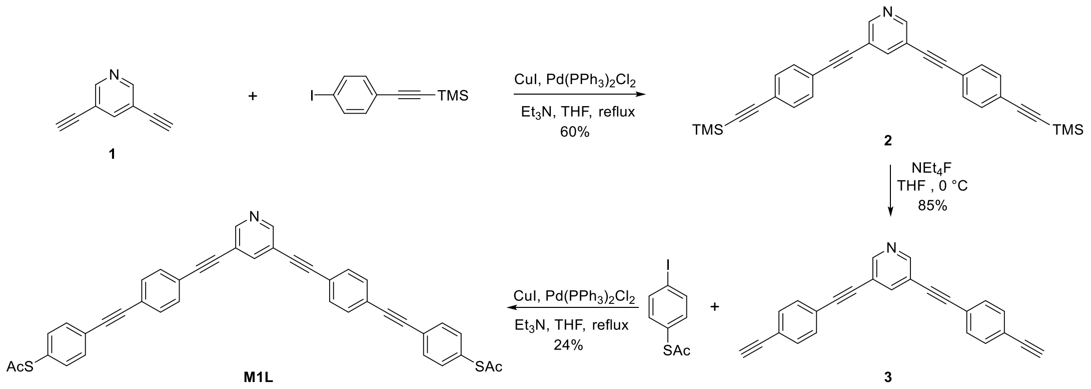

Figure S28. The synthetic route of **M1L**.

3,5-bis((4-((trimethylsilyl)ethynyl)phenyl)ethynyl)pyridine (Compound **2**): To a solution of 3,5-diethynylpyridine (200 mg, 1.57 mmol, 1 equiv.), ((4-iodophenyl)ethynyl)trimethylsilane (992 mg, 3.30 mmol, 2.1 equiv.) and 10 mL degassed Et_3N in 20.0 mL of anhydrous THF were added $Pd(PPh_3)_2Cl_2$ (56 mg, 0.08 mmol, 0.05 equiv.) and CuI (30 mg, 0.16 mmol, 0.1 equiv.) under an N_2 atmosphere. The reaction mixture was refluxed for 8 hours and then cooled to room temperature. Saturated ammonium chloride (10mL) was added, the resulting mixture was extracted with Et_2O (2 x 20 mL). The combined organic solution was washed with brine (20 mL), dried over anhydrous $MgSO_4$, filtered and concentrated under reduced pressure. The residue was purified by flash column chromatography to give compound 2 in 60% yield.

Characterization of compound **2**: Pale yellow solid. ^{1}H NMR (500 MHz, $CDCl_3$) δ 8.672 (d, J = 1.9 Hz, 2H), 7.914 (t, *J* = 1.9 Hz, 1H), 7.469 (s, 8H), 0.262 (s, 18H); ^{13}C NMR (125 MHz, $CDCl_3$) δ 150.9, 140.5, 132.0, 131.5, 123.8, 122.2, 119.9, 104.4, 96.9, 92.9, 86.9, –0.12. IR (neat, cm^{-1}): 3368, 3294, 2923, 2852, 1655, 1261, 1017, 836, 801, 750, 698, 670, 660, 428. HRMS (ESI): *m/z* calcd for $[C_{31}H_{29}NSi_2+H]^+$, 472.1911; found, 472.1935.

3,5-bis((4-ethynylphenyl)ethynyl)pyridine (compound **3**): The solution of compound **2** (200 g, 0.42 mmol) in THF (50mL) was added tetrabutylammonium fluoride (0.42 mmol, 1 M in THF) at 0 ^{o}C. The mixture was stirred for 15 min and saturated ammonium chloride (10mL) was added. The resulting mixture was extracted with Et_2O (2 x 20 mL). The combined organic solution was washed with brine (20 mL), dried over anhydrous $MgSO_4$,

filtered and concentrated under reduced pressure. The residue was chromatographed to give compound **3** in 85% yield.

Characterization of compound **3**: Brown solid. ^{1}H NMR (500 MHz, $CDCl_3$) δ 8.685 (s, 2H), 7.934 (s, 1H), 7.496 (s, 8H), 3.202 (s, 2H); ^{13}C NMR (125 MHz, $CDCl_3$) δ 151.0, 140.6, 132.2, 131.6, 122.8, 122.7, 119.9, 92.7, 87.0, 83.0, 79.3. IR (neat, cm^{-1}): 3358, 3206, 3029, 2957, 2922, 2851, 2155, 1633, 1259, 1134, 1087, 865, 836, 801, 698, 636, 485, 449. HRMS (ESI): *m/z* calcd for $[C_{25}H_{13}N+H]^+$, 328.1121; found, 328.1128.

S,S'-((((pyridine-3,5-diylbis(ethyne-2,1-diyl))bis(4,1-phenylene))bis(ethyne-2,1-diyl))bis(4,1-phenylene)) (compound **M1L**): To a solution of compound **3** (100 mg, 0.30 mmol, 1 equiv.), S-(4-iodophenyl) ethanethioate (175 mg, 0.63 mmol, 2.1 equiv.) and 5 mL degassed Et_3N in 10.0 mL of anhydrous THF were added $Pd(PPh_3)_2Cl_2$ (9 mg, 0.015 mmol, 0.05 equiv.) and CuI (5 mg, 0.16 mmol, 0.1 equiv.) under an $\mathbf{N_2}$ atmosphere. The reaction mixture was refluxed for 24 hours and then cooled to room temperature. Saturated ammonium chloride (10mL) was added, the resulting mixture was extracted with Et_2O (2 x 20 mL). The combined organic solution was washed with brine (20 mL), dried over anhydrous $MgSO_4$, filtered and concentrated under reduced pressure. The residue was purified by flash column chromatography to give compound **M1L** in 24% yield.

Characterization of compound **M1L**: Pale yellow solid. ^{1}H NMR (500 MHz, CD_2Cl_2) δ 8.676 (s, 2H), 7.953 (s, 1H), 7.53–7.58 (m, 12H), 7.412 (d, J = 8.0, 4H), 2.409 (s, 6H); owing to poor solubility of **M1L**, its ^{13}C NMR is difficult to be characterized. IR (neat, cm^{-1}): 3367, 2960, 2922, 2852, 1655, 1262, 1084, 1015, 837, 827, 796, 729, 696, 670, 636, 599, 554, 424. HRMS (ESI): *m/z* calcd for $[C_{41}H_{25}NO_2S_2+H]^+$, 628.1399; found, 628.1419

3,5-bis((4-(acetylthio)phenyl)ethynyl)-1-methylpyridin-1-ium trifluoromethanesulfonate (compound **M1-Me**): A mixture of compound **M1** (50 mg, 0.12 mmol) and methyl trifluoromethanesulfonate (30 mg, 0.18 mmol) in 10 mL diethyl ether was stirred at RT for 15 min to give a yellow precipitate, and then the precipitate was isolated by filtration. Further recrystallizing from the mixed solvent (DCM/Et_2O, 10/1) and evaporating under vacuum to give a pale yellow solid. Yield, 55 mg, 79%.

Characterization of compound **M1-Me**: Pale yellow solid, 79% yield. ^{1}H NMR (400 MHz, $CDCl_3$): δ = 8.86 (s, 2H), 8.49 (s, 1H), 7.63 (d, J = 8.29 Hz, 4H), 7.47 (d, J = 8.29 Hz, 4H), 4.43 (s, 3H) and 2.45 (s, 6H). ^{13}C NMR (100 MHz, CD_2Cl_2): δ = 193.10, 147.55, 145.51, 134.48, 132.66, 131.49, 125.03, 121.38, 98.12, 82.57, 49.19 and 30.25. IR (neat, cm^{-1}): 3369, 2923, 2853, 2218, 1658, 1632, 1467, 1412, 1350, 1265, 1084, 1031, 946, 897, 736, 700, 511. HRMS (ESI): *m/z* calcd for $[C_{26}H_{20}NO_2S_2]^+$, 442.0930; found, 442.0931.

Section 2. MCBJ Experimental Methods

Home-made MCBJ setup

As shown in Figure S11, our home-made MCBJ setup was used to measure the molecular conductance between two gold electrodes. During the MCBJ measurement, a sheet of steel was taken as substrate and fixed by two supports on both ends. The notched gold wire and liquid cell were fixed upon the substrate. The pushing rod below the substrate was used to bend and release the substrate repeatedly, resulting in the repeating breaking and re-connecting of gold wire. To have precise control, a piezo actuator was used as the pushing rod. During the repeating breaking and re-connecting operation, the real-time conductance was recorded by the home-built *I-V* converter.

Single-molecule conductance measurement

Conductance measurements were performed in solution at room temperature with our home-built MCBJ setup (Li et al., 2017). We used two kinds of solutions: a mixture of dichloromethane (DCM) / trichlorobenzene (TCB) (*v*/*v*: 1 / 4); pure propylene carbonate (PC). Protonated pyridiniums **M1-H**, **R1-H**, **R2-H**, **R3-H**, and **M1L-H** were in-situ formed according to published protocol (Boyle et al., 2017). The methylated pyridinium **M1-Me** are directly dissolved in corresponding solutions. In both solutions, we dissolved 1 mM of target molecules for the following characterizations. During the MCBJ measurement, a spring steel sheet was taken as substrate and fixed by two supports on both ends. The notched gold wire and liquid cell were fixed upon the substrate. The real-time conductance was recorded by the home-built *I-V* converter with a sampling rate of 20 *k*Hz. For each experiment, more than one thousand conductance traces were recorded for statistical analysis.

Data analysis

In the measurement of single-molecule conductance, since the variation of molecular junction configuration results the variation of conductance, in each experiment, we record thousands of individual single traces, and apply a statistical approach to determine the most probable conductance and the stretching distance.

The conductance traces without forming molecular junctions were excluded for analysis as reported in our previous paper (Hong et al., 2012, Huang et al., 2015, Liu et al., 2017a). One-dimensional conductance histograms were constructed by collecting all individual traces with a bin size of 1100 for log(G/G_0) from –10 to +1, and 1000 for Δz from –0.5 to 3 nm. The conductance distribution was extracted by calculating the data density in each bin. The peak shift in a conductance histogram was determined by Gaussian fitting, which represents the most probable molecular conductance.

2D conductance-displacement histograms were plotted by overlapping each individual trace with a bin size of 1100 for log(G/G_0) from –10 to +1, and 1000 for Δz from –0.5 to 3 nm. All traces are aligned with a relative zero point (Δz = 0) at G = 0.5 G_0. Then the 2D conductance distribution versus the relative distance was constructed by the data counts in each bin.

To construct the displacement distribution histograms, firstly the relative stretching distance, Δz, was determined from the position where the conductance is 0.5 G_0 (after the rupture of the gold-gold atomic break at G_0), to the molecular conductance region, just before the end of the molecular plateau. The peak represents the most probable plateau length. To find the absolute displacement, z^*, which is related to the most probable length of the molecular junction, the relative displacements were corrected by adding the snap-back distance, Δz_{corr}, to the relative displacement Δz, namely, $z^* = \Delta z + \Delta z_{corr}$. Referring to the previous result[3], Δz_{corr} was determined experimentally to be 0.5 ± 0.1 nm.

Evaluation of the concentration effect

When we change both the concentration of **M1** and TFA in MCBJ measurements (Figure S13), we find that the concentration chaning leads to the variation of junction formation probability for both connectivities. So that in our experiments, we use a relatively higher concentration (1.0 mM) to ensure high junction formation probability for both two connectivities.

Section 3. Junction Geometry Analyzing

Flicker noise analysing for the high-conductance junctions of M1-H

To probe the junction geometry, we performed flicker noise analysis on the high-conductance junction of **M1-H**. According to previous reports, the flicker noise of the single-molecule junctions reflects the coupling between electrodes and molecules (Adak et al., 2015, Garner et al., 2018). The noise power of through-space coupling scales as $G^{2.0}$, where G is the mean conductance. It is in contrast to the through-bond coupling, where the noise power scales as $G^{1.0}$. We paused the junction elongation process at the high-conductance region of **M1-H** for 150 ms, during which time the conductance signals were extracted out for noise analysis. As shown in Figure S14A, when the noise power is normalized by $G^{1.0}$, we find a positive correlation between the noise power and average conductance, and such correlation is minimized when the noise power normalized by $G^{1.8}$ (Figure S14B). Such correlation suggests a junction geometry of through-space coupling, which is consistent with the ring coupling of pyridinium to the gold electrode.

I/V characterization for the high-conductance junctions of M1-H

The I/V characterization was performed as the following: once a molecule junction was formed during the stretching process, we suspended the stretching process and applied a voltage ramp between -1 and 1 V. The ring coupling of **M1-H** would lead to an asymmetric junction geometry, which was verified by the I/V characterization, showing a moderately asymmetric character with a rectification ratio of ~1.5 (Figure S15).

Evaluating the bonding energy of Au-S through Surface enhance Raman spectra

The 0.1mM solution of target molecules in THF were dropped on the gold substrate to form molecule assembly and the gold nanoparticles dispersed in water were dropped and dried under vacuum by the water pump to form a ‘coffee ring’ pattern. The SERS spectra were collected at IDSpec Arctic system under 50X-long working

distance objective lens. The protonation was performed by adding 100 eq. TFA into the THF solution of target molecules, which were used for SERS characterization as mentioned above.

Characterization of reference molecules with one –SAc anchor

We characterized the single-molecule conductance of other pyridiniums **R1-H** (Figure S16A) with only –SAc group. We observe the formation of molecule junctions in the 1-D conductance histogram of **R1-H** (Figure S16B). The molecular conductance peak of **R1-H** centers at $10^{-3.2}$ G_0, which is distinct to the neutral state of **R1-H** with a conductance peak centers at $10^{-3.9}$ (Liu et al., 2017b). Meanwhile, **R1-H** shows a similar stretching pattern (Figure S16C) to **M1-H** with about 0.31 nm stretching distance. By characterizing another two reference molecules similar to **R1-H** with nitrogen set in different positions (Supplementary Figure S16), we also observe similar conductance and stretching patterns.

M1-Me (Figure S17A) is also able to form molecular junctions, with a molecular conductance of $10^{-3.6}$ G_0 (Figure S17B) and 0.35 nm junction stretching distance (Figure S17C), both of which is similar to **M1-H**.

Section 4. Reversible Switching

Calculating the junction formation probability of M1-H in different bias

By increasing the bias from 0.05 to 0.40 V gradually, we recorded about 2000 conductance traces of **M1-H** without data selection. As shown in Figure S18, we applied double peaks Gaussian fitting to both the high- and low-conductance junction, the corresponding junction formation probability are shown beside.

Reversible changing the junction formation of M1-H by switching the bias between 0.10 and 0.40 V

By varying the bias between 0.10 and 0.40 V alternatively, we recorded about 1000 conductance traces of **M1-H** without data selection. As shown in Figure S19, we applied double peaks Gaussian fitting to both the high- and low-conductance junction, the corresponding junction formation probability are shown beside.

The acid-base pair of M1 and M1-H with reversible conductance transformation

The MCBJ conductance measurement was firstly performed on the 1 mM solution (DCM/TCB = 1/4) of **M1**. After collecting more than one thousand conductance traces, the solution in the MCBJ setup was took out. Then 100 eq. TFA was added into the 0.1 mM solution of **M1**, and 50 μL of the mixed solution was put in the same MCBJ setup and the conductance traces were also collected for more than one thousand**.** The neutral pyridine was reproduced by the treatment of Na_2CO_3 aqueous solution (2.0 M) to the equal volume solution of **M1-H** (the mixed solution just formed), after mixing the solutions and stewing for stratification, the organic layer was taken out for conductance measurements. Circularly, this organic solution is treated again with TFA and Na_2CO_3 sequentially and all the single-molecule conductance measurements in each repetitive process are characterized with at least one thousand traces.

Section 5. Theoretical Calculation

Geometry optimizing for calculating junction lengths

It's worth noting that in the above experiment, especially in the acid condition, the thioacetates would be easily deprotonated at the gold surface (Lau et al., 2006), we used actually the deprotected analogies of **M1**, **M1-H**, **M1L** and **M1L-H** for the following calculations. All the calculations were performed with the Gaussian 09 software package(Frisch et al., 2013). The B3LYP/6-311++G(d,p)(Becke, 1993, Ernzerhof and Scuseria, 1999) level of density functional theory was used to optimize all of the structures for **M1** and **M1L** and the cation of **M1-H** and **M1L-H**. The theoretical junction lengths were calculated by the distance between two thiol groups or between one of the thiol and the middle point of pyridine rings.

Evaluating the bonding energy of Au-S

To evaluate bonding energy, the models were optimized by PBE0(Hay and Wadt, 1985a, b) method including dispersion correction (DFT-D3) (Grimme et al., 2010). Standard basic set 6-311G(d,p) was used to describe C, N, H and S atoms, and the effective core potentials. Lanl2TZ(f) was used to describe the effective core potential of Au(Hay and Wadt, 1985a, b, Wadt and Hay, 1985).

There are three effects to weaken the Au-S bond. Firstly, the Au-S covalent bond and Au←SH coordination bond will both be possible existing in the gold surface (Inkpen et al., 2019). And the **M1-H** is formed in situ from the acidic environment, which will inhibit the deprotonation of –SH, and facilitate the formation of Au←SH coordination bonds, with significantly weaker bonding energy (Figure S24). Secondly, according to hard/soft acid/base principles, gold is soft acid, which will have a stronger interaction with more polarizable atom. From the atomic charge distribution (Figure S26), the negative charge of sulfur atoms in **M1-H** is obviously smaller than that in **M1**, so that the sulfur atoms in **M1** is more polarizable than that in **M1-H**, which will lead to different bonding energy between them. Thirdly, the LUMO of **M1-H** is localized in the pyridinium ring, and thiol atoms show almost none distribution at the thiol atoms in the LUMO **M1-H**. While the LUMO of **M1** shows a delocalized pattern, the non-hybridized p orbital of the thiol atoms can conjugate with the π system, which will facilitate the back donation from the d orbital of golds to the p orbitals of thiols. The enhanced back donation will also lead to stronger Au-S bonding energy in **M1** than that in **M1-H**.

The effect of EEF on total electronic energy

To evaluate the effect of EEF, the models used to evaluate Au-S formation energy (Figure S24) were used to study their total electronic energy changes by altering the strength and orientation of EEF. The applied EEF was along the z-axis with the strength changing from –0.006 to +0.006 a.u., and the strength was fixed at +0.006 a.u with molecule orientation changed from -90° to 90°. Then all the models were optimized by PBE0 method including dispersion correction (DFT-D3) (Grimme et al., 2010). Standard basic set 6–311G(d,p) was used to describe C, N, H and S atoms, and the effective core potentials. Lanl2TZ(f) was used to describe the effective core potential of Au(Hay and Wadt, 1985a, b, Wadt and Hay, 1985).

Supplemental References

Adak, O., Rosenthal, E., Meisner, J., Andrade, E.F., Pasupathy, A.N., Nuckolls, C., Hybertsen, M.S., and Venkataraman, L. (2015). Flicker noise as a probe of electronic interaction at metal–single molecule interfaces. Nano Lett. 15, 4143-4149.

Becke, A.D. (1993). Density-functional thermochemistry. Iii. The role of exact exchange. J. Chem. Phys. 98, 5648-5652.

Boyle, T.J., Yonemoto, D.T., Sears, J.M., Treadwell, L.J., Bell, N.S., Cramer, R.E., Neville, M.L., Stillman, G.A.K., and Bingham, S.P. (2017). Synthesis, characterization, and utility of trifluoroacetic acid lanthanide precursors for production of varied phase fluorinated lanthanide nanomaterials. Polyhedron 131, 59-73.

Ernzerhof, M., and Scuseria, G.E. (1999). Assessment of the perdew–burke–ernzerhof exchange-correlation functional. J. Chem. Phys. 110, 5029-5036.

Frisch, M., Trucks, G., Schlegel, H.B., Scuseria, G., Robb, M., Cheeseman, J., Scalmani, G., Barone, V., Mennucci, B., and Petersson, G. (2013). Gaussian 09, revision d. 01 (Gaussian, Inc., Wallingford CT).

Garner, M.H., Li, H., Chen, Y., Su, T.A., Shangguan, Z., Paley, D.W., Liu, T., Ng, F., Li, H., Xiao, S., et al. (2018). Comprehensive suppression of single-molecule conductance using destructive σ-interference. Nature 558, 415-419.

Grimme, S., Antony, J., Ehrlich, S., and Krieg, H. (2010). A consistent and accurate ab initio parametrization of density functional dispersion correction (dft-d) for the 94 elements h-pu. J. Chem. Phys. 132, 154104.

Hay, P.J., and Wadt, W.R. (1985a). Ab initio effective core potentials for molecular calculations. Potentials for k to au including the outermost core orbitals. J. Chem. Phys. 82, 299-310.

Hay, P.J., and Wadt, W.R. (1985b). Ab initio effective core potentials for molecular calculations. Potentials for the transition metal atoms sc to hg. J. Chem. Phys. 82, 270-283.

Hong, W., Manrique, D.Z., Moreno-Garcia, P., Gulcur, M., Mishchenko, A., Lambert, C.J., Bryce, M.R., and Wandlowski, T. (2012). Single molecular conductance of tolanes: Experimental and theoretical study on the junction evolution dependent on the anchoring group. J. Am. Chem. Soc. 134, 2292-2304.

Huang, C., Rudnev, A.V., Hong, W., and Wandlowski, T. (2015). Break junction under electrochemical gating: Testbed for single-molecule electronics. Chem. Soc. Rev. 44, 889-901.

Inkpen, M.S., Liu, Z.F., Li, H., Campos, L.M., Neaton, J.B., and Venkataraman, L. (2019). Non-chemisorbed gold–sulfur binding prevails in self-assembled monolayers. Nat. Chem. 11, 351-358.

Lau, K.H.A., Huang, C., Yakovlev, N., Chen, Z.K., and O'Shea, S.J. (2006). Direct adsorption and monolayer self-assembly of acetyl-protected dithiols. Langmuir 22, 2968-2971.

Li, R., Lu, Z., Cai, Y., Jiang, F., Tang, C., Chen, Z., Zheng, J., Pi, J., Zhang, R., Liu, J., et al. (2017). Switching of charge transport pathways via delocalization changes in single-molecule metallacycles junctions. J. Am. Chem. Soc. 139, 14344-14347.

Liu, J.Y., Zhao, X.T., Al-Galiby, Q., Huang, X.Y., Zheng, J.T., Li, R.H., Huang, C.C., Yang, Y., Shi, J., Manrique, D.Z., et al. (2017a). Radical-enhanced charge transport in single-molecule phenothiazine electrical junctions. Angew. Chem. Int. Ed. 56, 13061-13065.

Liu, X., Sangtarash, S., Reber, D., Zhang, D., Sadeghi, H., Shi, J., Xiao, Z.Y., Hong, W., Lambert, C.J., and Liu, S.X. (2017b). Gating of quantum interference in molecular junctions by heteroatom substitution. Angew. Chem. Int. Ed. 56, 173-176.

Wadt, W.R., and Hay, P.J. (1985). Ab initio effective core potentials for molecular calculations. Potentials for main group elements na to bi. J. Chem. Phys. 82, 284-298.
